# Disparities in Online Use Behaviours and Chinese Digital Inclusion: A 10-Year Comparison

**DOI:** 10.3390/ijerph191911937

**Published:** 2022-09-21

**Authors:** Xiao Yu, Shu Liu

**Affiliations:** Northeast Asian Research Centre, Northeast Asian Studies College, Jilin University, Changchun 130000, China

**Keywords:** internet use, digital inclusion, socioeconomic status, time effect, cohort effect

## Abstract

This study focuses on the disparities in Chinese online use behaviours (frequency and diversity) based on educational background and socioeconomic status over 10 years to reveal the Chinese digital inclusion process. We used the China Family Panel Studies (CFPS) 2010 and 2018 panels and considered the time and cohort effects separately. Ordinary least squares analysis revealed usage frequency. The generalized partial proportional odds model demonstrated participants’ prioritization of online study, work, entertainment, and social activity. The results show that the profile of the individuals with the most time spent online has changed from those with high education and income levels to mid-range education and income levels. Individuals with high education and income levels prefer to use the internet for studying and working. There are no clear preference differences between entertainment and social activities amongst most educational backgrounds and socioeconomic statuses. Regarding frequency of internet use, digital inclusion has spread downwards from the upper to the middle classes. Regarding diverse internet uses, upper-class individuals prefer to conduct capital-enhancing activities, and youth remain the main force for diverse online activities; however, over time, middle-aged groups have increased their capital-enhancing activities, and older adults have increased their digital social activities.

## 1. Introduction

The use of information and communication technology (ICT) has been acknowledged as the third life skill after literacy and numeracy [1]. Digital technologies have significantly affected individuals’ choices, improved quality of life, and enhanced social inclusion, especially for older adults [2]. Meanwhile, people with high digital skills and diverse ICT usage are described as the information elite [3], and digital inclusion outcomes include civic engagement, policy influence, social change, and economic advancement [4]. In China, the internet penetration rate increased from 28.9% in 2009 to 70.4% in 2020. Since 2009, the characteristics of netizens have gradually changed in terms of age, from youth (aged 10–29 years) to middle-aged adults; and socioeconomic backgrounds from high to relatively low educational backgrounds and income levels [5,6].

To meet people’s needs and drive high-quality economic development, China has implemented *Digital China* as its information construction plan covering fields such as economics, politics, culture, society, and ecology to provide useful, affordable, and well-used information services [7]. Useful services emphasise the popularisation of information services and infrastructure construction. Affordable services emphasise the reduction of application costs and network fees. Between 2016–2021, the average fixed broadband bandwidth and mobile network traffic rates decreased by more than 95% [8]. Well-used services emphasise network speed (to increase effective internet access and use) and network diversity (e.g., to combine education, medical services, digital governance, and other aspects) to enhance user satisfaction. In the third quarter of 2017, users’ fixed broadband weighted average download rate was 16.4 Mbit/s during busy and idle hours across China, which was a year-on-year increase of 48.7% [7].

Accordingly, China, as a fast-growing economy, has increased the prosperity of the entire population and the proportion of the relative wealthy middle class. However, economic inequalities have also increased. One reason is that the economic benefits of ICT use are first distributed to people who already have high socioeconomic status [9]. While access to information can initially solve inequality, such access is currently presented as involving upwards inequality, polarisation, and social exclusion [10]. Furthermore, the uneven distribution and application of digital resources have led to a digital divide.

The Organisation for Economic Co-operation and Development (OECD) [11] defines the digital divide as ‘the difference in the accessibility of ICT and the use of the internet among individuals, families, enterprises, and regions of different socioeconomic status’ (p. 5). The divide is categorised into the first gap (i.e., access to the internet), second gap (i.e., differences in internet use), and third gap (i.e., outcomes and consequences of internet use). While the access gap has been closed in many countries, the skills gap is increasing; therefore, the research focus has shifted from the access gap to the usage gap [10,12,13,14,15].

The digital divide leads to exclusion that can be considered costly because it encompasses social, economic, and political spheres; for example, through academic failure, unemployment, low productivity, and social isolation [1]. Moreover, the exacerbation of existing social inequalities is attributed to the differences in internet use: the unequal distribution of digital skills due to different social, psychological, and cultural backgrounds; and the economic and social impacts of technology [15,16,17]. Thus, it is essential to understand the digital divide as a political and social issue rather than a technological one [13]. Indeed, most researchers accept that the digital divide exists, and the reliance alone on innovations and markets cannot solve it [18].

Regarding the disparities in ICT use, individuals conduct different online activities to enhance their human, financial, political, social, and cultural capital. Meanwhile, some capital-enhancing activities, such as study and work, can help individuals achieve upwards mobility [19]. Therefore, based on the second digital divide (i.e., differences in internet use), this study focuses on the disparities in Chinese netizens’ online use behaviours based on their educational backgrounds and socioeconomic statuses over 10 years. This study differentiates internet use by its depth of use (i.e., frequency of use) and width of use (i.e., diversity of online activities), and uses data from the China Family Panel Studies (CFPS) 2010 and 2018 panels. This study explores the in-depth disparities using an ordinary least squares (OLS) analysis. A generalised partial proportional odds model is used to detect participants’ evaluations of the importance of four online activities: studying, working, entertainment, and social activity. The width of internet use is explored by separately addressing the time and cohort effects, as follows. First, based on the national-level inference characteristic of the CFPS, the time effect explores the impact of participants’ different educational backgrounds and socioeconomic statuses on the depth and width of their internet use in the two panels. The time effect analysis reveals the national change in online use behaviours and development of the digital inclusion process over 10 years. Second, the cohort effect explores the changes in the participants’ evaluations of the four online activities across the two panels, arranged by birth year, to observe the digital inclusion process over 10 years. Finally, this study conducts a correlation analysis between the participants’ online activity evaluations in the 2010 panel and their socioeconomic statuses in the 2018 panel to examine the association between internet use behaviours and outcomes.

## 2. Literature Review

### 2.1. Digital Inclusion/Exclusion and Their Relationship with Social Inclusion/Exclusion

The National Digital Inclusion Alliance (NDIA) [20] defines digital inclusion as ‘ensuring that all individuals and communities, including the most disadvantaged, have access to and use of ICT’. Digital inclusion has the following characteristics: (1) it ensures equal digital participation in and utilisation of available digital resources; (2) depending on willingness and capability, it helps individuals and communities to grasp efficient information and earn social benefits via ICT; and (3) it makes use of the extension capabilities of ICT to better serve society [16,18,21,22]. Digital inclusion also involves detailed measurements. For example, one study mentions it has ‘5Cs’: connectivity, capability, content, confidence, and continuity [16], while another provides five factors: affordable service, devices that meet users’ needs, digital literacy, quality technical support, and self-sufficiency content [20]. Accordingly, digital inclusion concerns the elimination of the barriers to the access and use of ICT [20].

Conversely, digital exclusion refers to the people who are slow to adopt ICT, and thus, are excluded from community participation via digital technologies [17]. Digital exclusion is the outcome of a lack of digital competence and information literacy [23].

Digital exclusion/inclusion is strongly associated with social exclusion/inclusion [15,18,24]. Social exclusion can lead to digital exclusion, as those who have already been socially excluded are more likely to be digitally excluded. Meanwhile, digital exclusion is rooted in social exclusion and exacerbates the existing social exclusion [17,25,26,27]. Some researchers suggest that digital exclusion is the consequence of social exclusion [25]. However, social exclusion does not necessarily result in digital exclusion, as digital access and usage can act as mediators [26]. For example, digital engagement can lessen the social exclusion condition, as social inclusion can be achieved through digital inclusion by efficient ICT usage [2,18,28]. Therefore, digital inclusion has become an essential factor for social inclusion [28,29]. However, the uneven distribution of ICT usage can lead to new forms of exclusion and inequality to form a negative correlation between ICT usage and social exclusion [30,31]. Therefore, the link between digital exclusion and social exclusion requires prioritising the solution to unequal ICT access and use [13], and macro-economic, meso-social, and micro-psychological factors should be considered to discover this relationship [26].

### 2.2. Digital/Social Inequality and Their Relationship

Exclusion and inequality can be interactively substituted [9,32], as the social divide between uneven resource distribution and socioeconomic categories results in inequality. Social inequality reflects the disparities in socioeconomic resources, knowledge, and physical ability, while digital inequality reflects the differences in ICT access and use followed by the capability, engagement, and usage outcomes of ICT [9,14,32]. Digital inequality is further differentiated as being absolute and comparative, whereby absolute inequality refers to the complete exclusion of people who have no ICT access or skills, and comparative inequality refers to people who receive greater or earlier benefits from ICT and can thus be regarded as beneficiaries [9].

Social inequality leads to digital inequality [4]; subsequently, the latter mirrors social inequality [29,33]. If digital resources are unevenly distributed, then these digital inequalities may reproduce, reinforce, and even exacerbate the existing social inequalities in ICT access and use, which, in turn, may enlarge the existing inequalities [9,12,15,34,35]. Digital inequality is increasing, and its relationship with other inequalities is still unclear [35]. Therefore, such inequality and other traditional inequalities should be addressed simultaneously, and digital inequality and digital exclusion should also be analysed in the offline context [35].

### 2.3. Factors Predicting ICT Access and Use

Youth are likely to possess more ICT skills [12,19,34], and there are gender differences in ICT engagement and skills [12,34]. Individuals who are equipped with hardware ownership and internet access transfer their educational attainments and knowledge into better ICT experiences [1,24]. For example, higher-educated individuals may own personal computers, have access to the internet at home, spend more time online, and conduct various types of online activities [12,19,29,34]. Higher-educated individuals may also be more skilled in conducting complex and purposeful ICT usage, while less-skilled individuals may prefer recreation-oriented ICT usage [24]. Furthermore, high internet skills and digital literacy can enhance the intensity of ICT use in online activities, and are also factors for digital inclusion [3,23,36]. ICT use is regarded as a skill, so ICT deficiency use will lead to exclusion in some societies [30]. However, educational attainment is not indicative of digital skill; for example, some undergraduates have high education levels but limited ICT skills [15].

Economic, social, cultural, and personal traits are essential factors for predicting ICT access and usage [17,37]. First, an individual’s social status shapes their interpretation of the benefits and consequences of digital media. Individuals with high socioeconomic status gain more benefits from the time spent online while conducting important ICT activities such as economic, political, and educational activities. Conversely, individuals with low socioeconomic statuses spend more time offline and use ICT less; therefore, they become digitally excluded [17,24,37]. Second, income, as a reflector of socioeconomic status, has a positive effect on ICT use [10,38]. For example, economic position can predict ICT usage in individual categories, as manual jobs (e.g., farming, fishing, and mining) and white-collar jobs require different levels of ICT engagement [33,34,35]. However, one study argued that younger, wealthier, and more educated individuals gain more digital benefits from ICT use, and their less-privileged counterparts find it difficult to catch up [21].

### 2.4. Hypotheses

Becker’s [39] human capital theory suggests that expenditure on education, training, and medical care is regarded as investing in capital. Earnings and productivity are increased through scientific and technical knowledge, skills, and problem-solving. Thus, knowledge growth can create more output. Digital hardware, software, and other digital resources work in the same way. Therefore, ICT use is an investment in human capital and knowledge and can be regarded as digital human capital that increases an individual’s welfare [37,38,40].

Furthermore, in the digitisation age, the transformation from digital resources to social resources (i.e., digital inclusion) relies on the interactions between digital capital and other capitals; the latter includes social, economic, personal, political, and cultural capital [21]. One study found that compared to economic and cultural capital, social and personal capital are more manageable and easily influenced by outside factors [25]. Therefore, the depth and width of ICT use should be considered when addressing digital inclusion and human capital. Regarding depth of use, this study considers the digital inclusion process for individuals with different socioeconomic statuses and posits the following:

**Hypothesis** **1A.** *Individuals with the highest education level spend more time online in the 2010 panel.*

**Hypothesis** **1B.** *Individuals with the highest income spend more time online in the 2010 panel.*

**Hypothesis** **1C.** *Compared with extreme educational cases, individuals with a middle- or high-school education spend more time online in the 2018 panel.*

**Hypothesis** **1D.** *Individuals with a middle income spend more time online in the 2018 panel.*

ICT usage cannot simply be distinguished as meaningful ICT use or digital engagement [10]; instead, it should be regarded as digital investment in human capital for the following reasons. First, while online activities are heterogeneous, some are regarded as capital-enhancing activities because they can add value and be transferable resources. Under the positive multiplied effects from these capital-enhancing activities, individuals are attracted to jobs, finances, health, and political participation, and gain social, financial, political, and cultural capital; thus, they are likely to increase their chances of upwards mobility [19,38,41]. For example, there is a usage gap between work and educational activities (which have capital-enhancing effects) and recreational activities (which do not) [19].

Second, individuals with different socioeconomic backgrounds present disparities in their intentions towards and quantities of ICT activities, leading to diversified outcomes of self-selection. Youth and adults with high educational backgrounds and socioeconomic statuses focus more on the information purposes of ICT use than individuals with low socioeconomic statuses [41]. This also applies to older adults, who are usually regarded as marginalised in terms of their ICT access and use. However, older adults with high educational backgrounds and socioeconomic statuses use the internet in beneficial and capital-enhancing ways [42], while disadvantaged households are more likely to use ICT for entertainment purposes [31].

Therefore, regarding the width of internet use, this study posits the following:

**Hypothesis** **2A.** *Individuals with high educational backgrounds and socioeconomic statuses prefer to conduct capital-enhancing activities on the internet (e.g., studying and working) than people with low educational backgrounds and socioeconomic statuses.*

**Hypothesis** **2B.** *Individuals with low educational backgrounds and socioeconomic statuses prefer to conduct recreational activities on the internet (e.g., entertainment and social activities) than people with high educational backgrounds and socioeconomic statuses.*

## 3. Materials and Methods

### 3.1. Methods

#### 3.1.1. OLS Model

This study constructed an OLS model to test the differences in the depth of internet use in the 2010 and 2018 panels. The model is:

(1) Internet time=α+βX+e

where the dependent variable is an individual’s time spent online, *X* is the independent variable, α and β are the coefficients, and *e* is the error term. Robust cluster standard errors are supplied to control for the CFPS’s regional disparities (25 regions in the 2010 panel and 31 regions in the 2018 panel).

#### 3.1.2. Generalised Partial Proportional Odds Model

Online activity importance was evaluated using a five-ranked categorical variable that included studying, working, entertainment, and social activities. The study adopted the following generalised partial proportional odds model to measure the effects:

(2) Study activity evaluation=α+βX+e

(3) Work activity evaluation=α+βX+e

(4) Entertainment activity evaluation=α+βX+e

(5) Social activity evaluation=α+βX+e 

where the dependent variable is the online activity evaluation, *X* is the independent variable, α and β are the coefficients, and *e* is the error term. Robust cluster standard errors are supplied to control for the CFPS’s aforementioned regional disparities.

Due to the ordered characteristics of the dependent variable, this study first conducted an ordered logit model, but then rejected it because it violated the parallel lines assumption via the Brant test. Normally, if the parallel lines assumptions hold, all coefficients (except the intercepts) will be the same across equations, except for the sampling variability [43]. Therefore, this study adopted the gologit2 programme to conduct a generalised ordered logit model. Gologit2 is less restrictive towards the model assumptions and more interpretable than the ordered and multinomial logistic models. Furthermore, this study applied the autofit option to the model so as to relax the specific variables, which were not justified and violated the parallel lines assumptions; this was done in order not to set all variables free from the parallel lines’ assumptions [43]. This study then used the *coefplot* Stata add-on to make the coefficients easy to read [44]. Accordingly, the coefficients can be interpreted as follows: if the coefficient is positive, the participant is more likely to move into a higher category; if the coefficient is negative, the participant is likely to either remain in the current category or move into a lower category.

#### 3.1.3. Robustness Test

This study focuses on the effects of educational backgrounds and socioeconomic statuses on online activity evaluations. The study used the International Socioeconomic Index of Occupational Status (ISEI) scores to determine an individual’s educational background and socioeconomic status to test the robustness of the model. Consequently, the observations in the samples were shrunk.

#### 3.1.4. Correlation Analysis

The third level of the digital divide concentrates on the consequences and outcomes from internet usage. Although this study focuses on the disparities in individual online use behaviours in the depth and width of internet usage, we also discovered an association between the importance of the online activities in the 2010 panel and socioeconomic status in the 2018 panel. Therefore, we conducted a correlation analysis to examine the association among the variables. The model is:

(6) $$r=\frac{1}{n-1}\sum_{i=1}^{n}\left(\frac{x_{i}-\bar{x}}{s_{X}}\right)\left(\frac{y_{i}-\bar{y}}{s_{Y}}\right)$$

where *X* represents the four online activities (study, work, entertainment, and social activity) in the 2010 panel, $\bar{x}$ and $s_{X}$ are the means and standard deviations, *Y* is the ISEI scores in the 2018 panel, and $\bar{y}$ and $s_{Y}$ are the mean and standard deviation. The significance of the association is supplied using asterisks.

### 3.2. Data

This study used the CFPS data from the 2010 baseline panel and 2018 panel. The CFPS collects data from individuals, families, and communities to reflect the changes in China’s society, economy, population, education, and health. It is funded by Peking University and the National Natural Science Foundation of China and maintained by the Institute of Social Science Survey of Peking University [45,46]. The CFPS provides a national representative sample because the population of all provinces in which participants are located covers 94.5% of the total population of China in the 2010 panel [46].

To analyse the depth and width of internet usage, this study retained the netizens in the 2010 and 2018 panels, which was attributed to 18.68% and 46.86% of the original datasets in the 2010 and 2018 panels, respectively. In total, 6278 internet users participated in the 2010 panel and 17,505 in the 2018 panel. This study merged the two panels into one sample (totalling 20,728 individuals) to further test the time effect of online use behaviours. In our study, 3055 individuals were represented across both panels. After checking for gender and age, 3042 individuals qualified to test the cohort effect of differences in online use behaviours.

This study included two separate samples for the online activity evaluations. First, the time effect sample focused on individuals’ evaluation of the importance of specific online activities (studying, working, entertainment, and social) in the 2010 and 2018 panels; these outcomes can reflect the national performance of digital inclusion in related years. Second, the cohort effect sample focused on individuals who took part in interviews in both the 2010 and 2018 panels to present the digital inclusion process through the same participants but with different online use behaviours among the two panels.

### 3.3. Variables

#### 3.3.1. Explained Variables

Internet time. This included the time spent on the internet. The responses to questions in the 2010 panel (e.g., ‘In the most recent non-holiday month, how many hours per day approximately constitute your average daily time spent online?’) and the 2018 panel (e.g., ‘Under normal circumstances, how many hours do you spend online in your spare time each week?’) were measured numerically. The missing values were transformed to null.

Online activity evaluation. This included the responses to questions such as ‘How important are studying/working/entertainment/social online activities for you when using the internet?’ The responses were ranked as 1 (no importance), 2 (low importance), 3 (neutral), 4 (some importance), 5 (utmost importance). Regarding the online activities, ‘studying’ referred to searching for study materials and taking online classes. ‘Social activity’ referred to online chatting, posting on Weibo, and so on. ‘Entertainment’ included watching online videos, downloading music, and so on.

The data-washing process included the following two special treatments for the missing data. First, in the 2018 panel, some values of the online activity evaluations were not applicable because the CFPS initially asked how frequently the participants took part in different online activities. If a participant answered ‘never’, then the online activity evaluations were marked as ‘not applicable.’ Therefore, this study transformed the ‘not applicable’ value as 1 (no importance) to remove the missing data. Second, when testing the cohort effect, the working online activity’s crosstab analysis revealed empty cells, which broke the presumptions for the generalised ordered logit model method. Therefore, when considering the cohort effect, the working online activity evaluation in the age cohort sample was measured using 1 (less importance), 2 (neutral), 3 (some importance) and 4 (utmost importance). In the five-ranked categorical variable for internet activity evaluation, the gologit2 method presented the four panels as tables and pictures, which included the 1st panel (category 1 versus categories 2, 3, 4, and 5); 2nd panel (categories 1 and 2 versus categories 3, 4, and 5); 3rd panel (categories 1, 2, and 3 versus categories 4 and 5); and 4th panel (categories 1, 2, 3, and 4 versus category 5).

#### 3.3.2. Explanatory Variables

Age range. Prensky [47] defines a digital native as an individual who grows up with technology, and a digital immigrant as an individual who learns new digital knowledge while retaining knowledge from the past. The internet appeared in China in 1995, so digital natives refers to those born after the 1980s, and digital immigrants refers to those born between the 1960s and 1970s [48]. Therefore, based on the sample weights, this study establishes four age ranges: less than 24 years, 25–31 years, 32–49 years, and more than 50 years. This variable applies to the time-effect sample.

Age cohort. In the cohort effect sample, the participants took part in the 2010 and 2018 panels. While age increases with years, the birth year remains a stable variable. Therefore, this study establishes four birth-year cohorts: before 1960, 1960–1978, 1979–1986, and 1987–1994. This variable applies to the cohort-effect sample.

Gender. Females are coded 1; males are coded null.

Urban. Participants who live in urban areas are coded 1; those who live in rural areas are coded null.

Education. This highest educational qualification variable has four categories: less than elementary school, middle school, high school, and beyond high school. ‘Less than elementary school’ includes illiteracy, no need for schooling, and level of elementary school, while ‘beyond high school’ includes college, bachelors, masters, and PhD attainment. This variable applies to the time effect sample.

Education in the cohort effect sample. Because of the sample weights, this highest educational qualification variable has three categories: less than middle school, high school, and beyond high school. This variable applies to the cohort effect sample.

Income status. The responses to the question ‘How do you measure your income status in the city in which you live?’ were ranked from 1 (low), 2 (lower middle), 3 (middle), 4 (higher middle), to 5 (high). The rankings of 4 and 5 are combined for high income; thus, there are four levels for income status.

Social status. The responses to the question ‘How do you measure your social status in the city in which you live?’ were ranked from 1 (low), 2 (lower middle), 3 (middle), 4 (higher middle), to 5 (high). The rankings of 4 and 5 are combined for high social status; thus, there are four levels for social status.

ISEI. The ISEI uses occupation as a reflection of education and income and combines various socioeconomic factors to obtain the scores; it also regards occupation as an objective rather than subjective status [49,50]. This study used the ISEI scores for the education and income variables and performed a robustness check. The occupation variable was supplied by the CFPS and obtained in two steps. First, the Chinese Standard Classification of Occupations (CSCO) was transformed into the International Standard Classification of Occupation (ISCO-88); second, the outcomes of the ISCO-88 were calculated as ISEI scores [51]. However, in the CFPS, the military, unemployed, and practitioner groups were inconvenient to classify; thus, they were not assigned to the ISCO-88, and regarded as missing values [51]. Based on the samples’ weighted scores, this study categorised the occupation variable as low, lower middle, middle, and high socioeconomic status to emphasise the difference effects and apply these categories in the robustness test.

## 4. Results

The descriptive statistical analysis in Table 1 has two parts: the time effect, which focuses on the internet use behaviours in the 2010 and 2018 panels to reflect the national trend of internet use for the specific years; and the cohort effect, which examines the changes in internet use behaviours over 10 years. Regarding the time spent online, the average was 2.131 h per day in the 2010 panel, and 13.540 h per week in the 2018 panel. As the samples are representative of the national trend for the specific years, the age weights of the time spent online showed older adults’ gradual entrance into the digital world in the 2018 panel. The education online weight changes are diversified, as participants with less than an elementary school education showed increased weights, while the weights of higher education (including high school and beyond high school) decreased, which partly reflects internet use being more available to people with a lower educational background. The average online activity evaluations for studying, working, entertainment, and social activity were higher than 3 in the 2010 panel, which shows that these activities hold a level of importance that is at least within the neutral importance category, if not more. Under the cohort effect, the age variable was unevenly distributed, and 6.66% of the participants were born before 1960. The number of urban people increased, while the weights of participants with extremely low levels of education, income, and social status decreased from the 2010 to the 2018 panel. Apart from the studying activity, the other three online activities’ average evaluations of importance increased, especially for the social activity, which showed a huge increase.

The time differences among the variables and independence test between the explanatory and explained variables (see Table A1 in Appendix A) were checked using a chi-square test. The results show, first, that the main explanatory variables and most of the explained variables had time differences; second, there were dependent relationships between the explanatory and explained variables.

Table 2 shows the depth of the internet usage results. Regarding time spent online, youth aged below 24 years spend more time online in both panels. Regarding educational level, the 2010 panel shows a clear gradient: those with higher educational levels spend more time online. In the 2018 panel, the middle school, high school, and beyond high school levels were significantly positive compared to the less than elementary school level; however, individuals with a high school education spent more time online than those with an education beyond high school. Those with a high income spent more time online than the other levels in the 2010 panel; however, individuals with a middle income spent more time online in the 2018 panel. Combined with educational level in the 2018 panel, the depth of internet usage partly reflects the downwards spread from upper class to middle class, and individuals with high social status spent significantly less time online.

**Figure 1:**
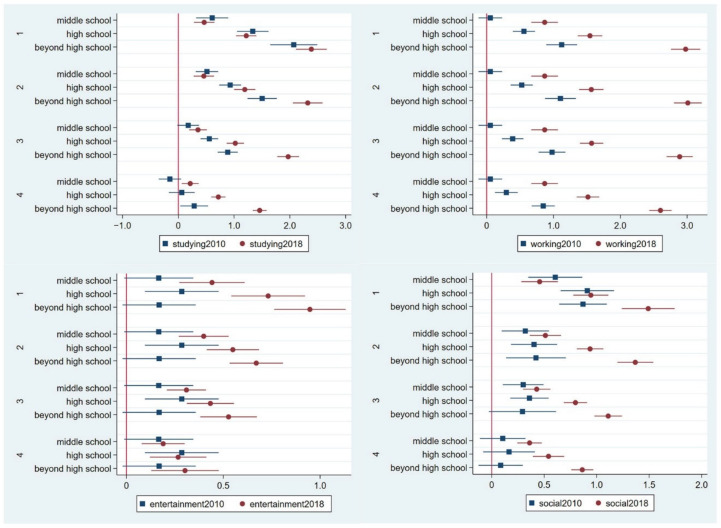
Results of educational background from the generalised partial proportional odds model predicting the importance of studying, working, entertainment, and social online activities in the 2010 and 2018 panels. Note: The four panels of internet activity evaluation included the 1st panel (category 1 versus categories 2, 3, 4, and 5); 2nd panel (categories 1 and 2 versus categories 3, 4, and 5); 3rd panel (categories 1, 2, and 3 versus categories 4 and 5); and 4th panel (categories 1, 2, 3, and 4 versus category 5). The detailed data are provided in Supplemental material Table S1.

Regarding the disparities in the socioeconomic statuses, this study focuses on the differences in the width of internet usage to measure the importance of four online activities: studying, working, entertainment, and social activity. Figure 1 shows the effects of education on these internet activities. First, comparisons among different educational attainments: the effect of education on the studying and working activities was almost positive in both panels, which shows that compared to individuals with a lower than elementary school education, individuals with higher educational attainment preferred to study and work online, as the coefficients increase in multiples. The effect of education was positive for the entertainment and social activities. In short, compared to those with a lower than elementary school education, those with higher educational attainment preferred to conduct entertainment and social activities online; however, in both panels, those with middle school, high school, and beyond high school education levels showed less variation. In the 2010 panel, individuals with a high school education were more likely to prefer conducting entertainment and social activities online than individuals with an education level beyond high school. Second, comparison among effect size: when comparing the coefficients in the 2010 and 2018 panels, the effect of education on the online activity evaluations was greater in 2018 than in 2010. Therefore, this study draws the conclusion that individuals with higher educational attainment preferred to use the internet for studying and working; however, preferences regarding entertainment and social activities did not clearly show any variation in educational attainment, except for individuals with extremely low education levels.

**Figure 2:**
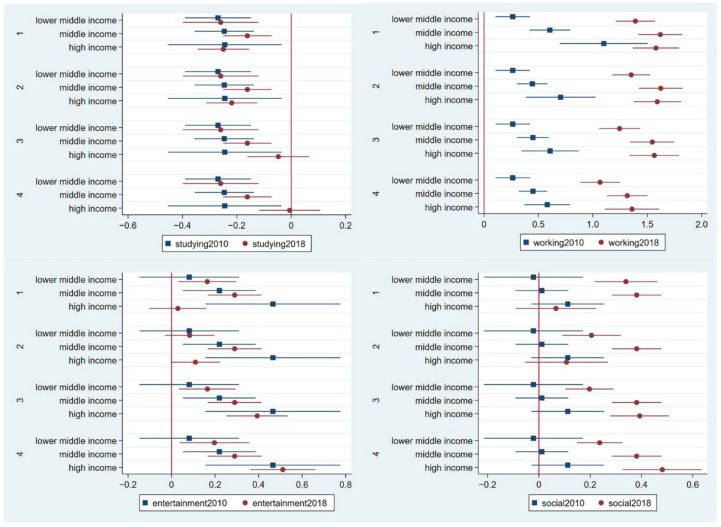
Results of income level from the generalised partial proportional odds model predicting the importance of studying, working, entertainment, and social online activities in the 2010 and 2018 panels. Note: The four panels of internet activity evaluation included the 1st panel (category 1 versus categories 2, 3, 4, and 5); 2nd panel (categories 1 and 2 versus categories 3, 4, and 5); 3rd panel (categories 1, 2, and 3 versus categories 4 and 5); and 4th panel (categories 1, 2, 3, and 4 versus category 5). The detailed data are provided in Supplemental Material Table S1.

**Figure 3:**
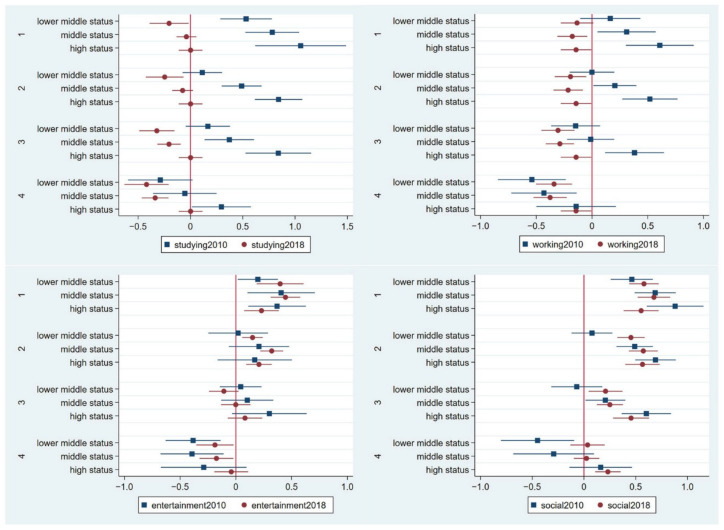
Results of the social status level from the generalised partial proportional odds model predicting the importance of studying, working, entertainment, and social online activities in the 2010 and 2018 panels. Note: The four panels of internet activity evaluation included 1st panel (category 1 versus categories 2, 3, 4, and 5); 2nd panel (categories 1 and 2 versus categories 3, 4, and 5); 3rd panel (categories 1, 2, and 3 versus categories 4 and 5); and 4th panel (categories 1, 2, 3, and 4 versus category 5). The detailed data are provided in Supplemental Material Table S1.

Figure 2 and Figure 3 show the socioeconomic status by income, property, and social standing. The correlation between income status and social status was 0.495 in the 2010 panel and 0.535 in the 2018 panel; thus, this study considers them complementary. First, regarding the studying online activity, nearly all the income status coefficients were negative in both panel years, which shows that income status is irrelevant for the studying activity. Individuals with the highest social status recognised the importance of studying online, but the data were insignificant in panel 2018. Second, regarding the working online activity, the coefficients of income status were all positive in both panels, and variations of the coefficients exist in the income status, except for the extremely low condition. Some of the social status coefficients were positive for individuals with the highest social status in the 2010 panel; however, all coefficients were negative in the 2018 panel. Therefore, individuals with a high-income status use the internet for working online. Third, regarding the entertainment online activity, individuals with a high income will rate the importance of conducting entertainment activities online higher, but there are not many variations seen in entertainment activities as an effect of variations in income. Individuals with high social statuses were more likely to score the importance of the entertainment online activity as neutral. The effect sizes in the 2010 and 2018 panels were similar. Forth, except for in extremely low situations, there are no clear variations towards the effect of the social online activity on individuals of different statuses. Therefore, individuals with a high income prefer to conduct work activities online; otherwise, no specific cohorts present strong preferences towards the entertainment and social online activities.

This study checked the conclusions via a robustness test; the results are shown in Table 3. The ISEI scores were substituted for education, income, and social status. The same conclusions were obtained from the robustness test; that is, individuals with higher ISEI scores preferred to conduct studying and working online activities, and no clear preference differences were shown among the individuals with different ISEI scores towards the entertainment and social online activities.

**Figure 4:**
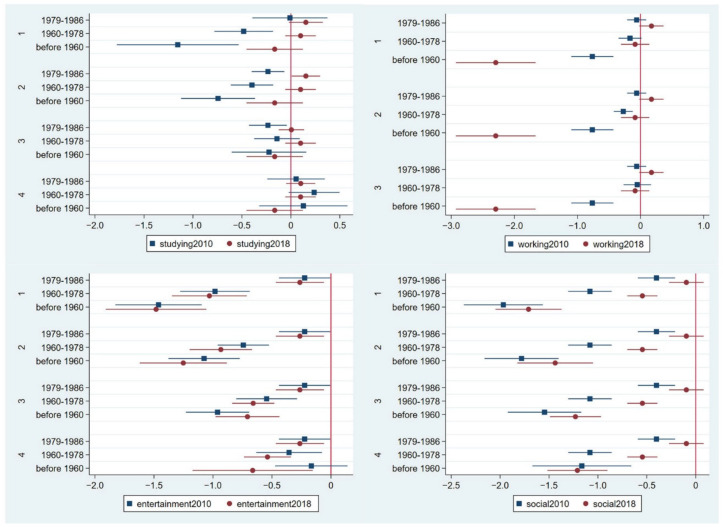
Results of the age cohort from the generalised partial proportional odds model predicting the importance of studying, working, entertainment, and social online activities in the 2010 and 2018 panels. Note: The four panels of studying, entertainment and social activity evaluation included the 1st panel (category 1 versus categories 2, 3, 4, and 5); 2nd panel (categories 1 and 2 versus categories 3, 4, and 5); 3rd panel (categories 1, 2, and 3 versus categories 4 and 5); and 4th panel (categories 1, 2, 3, and 4 versus category 5). Three panels of working activity evaluation included 1st panel (category 1 versus categories 2, 3, and 4); 2nd panel (categories 1 and 2 versus categories 3 and 4); 3rd panel (categories 1, 2, and 3 versus category 4). The detailed data are provided in Supplemental material Table S3.

This study focused on the cohort effect of the same birth year in the 2010 and 2018 panels to reveal the participants’ assessments of the four online activities. Figure 4 shows the results. In the 2010 panel, compared with those born between 1987–1994, the other three birth cohorts all had negative coefficients for online activities, which shows their low importance. Those born before 1960 abstained from using the internet to socialise, as the variations of the coefficients are large for the social online activity. However, the trend changes in the 2018 panel. For the studying online activity, the middle-aged cohorts (born between 1979–1986 and 1960–1978) showed positive coefficients. For the working online activity, the 1979–1986 cohort was positive. Furthermore, all three cohorts, except the youngest cohort, showed negative coefficients towards the entertainment online activity. Regarding the social online activity, all cohorts showed negative coefficients, except the youngest cohort; however, fewer variations were shown among the 1987–1994, 1979–1986, and 1960–1978 cohorts in the 2018 panel than in the 2010 panel, which partly reflects that these individuals have gradually accepted the internet and applied it to daily life as a way of being digitally included.

Under the cohort effect sample, the correlation test results in Table 4 show the association between the importance of the online activities in the 2010 panel and socioeconomic status in the 2018 panel to measure the outcomes of internet usage over time. Socioeconomic status is reflected by the ISEI scores. Although the correlation analysis does not differentiate between the explanatory and explained variables, in this study, the online activities in 2010 had occurred before the socioeconomic statuses were measured in 2018; thus, the association direction is unilateral from the 2010 panel to the 2018 panel.

This study performed a chi-square test to test the independence between the online activities in the 2010 panel and the ISEI scores in the 2018 panel. The results showed that all online behaviours and ISEI scores were co-dependent. There were significant positive linear correlations between the studying, working, and social online activities and the ISEI scores. The correlations were higher than 0.10 for the studying and working activities. The entertainment activity showed a negative but insignificant linear association with the ISEI scores, which insignificantly showed that individuals who perceive the entertainment activity as more important are likely to have a decreased socioeconomic status in the future.

## 5. Discussion

This study explored the transformation from digital resources to social resources (i.e., digital inclusion) through disparities in acceptance time, online activity preferences, and outcomes. The results are as follows. First, the disparities in acceptance time were shown through age and educational attainment. Individuals aged below 24 years spent more time online and placed great importance on studying, entertainment, and social online activities. The knowledge gap concept posits that when a new technology is presented, individuals with higher educational backgrounds and socioeconomic statuses will be the first to react, accept, and apply it in their daily lives; thus, the knowledge gap among cohorts will increase [52]. One study further argued that while new technologies supply learning resources to help people attain higher education, individuals who already have high education levels will be the first to benefit [17].

Therefore, setting the acceptance time earlier for the youth who live in remote countries and older people could help enhance digital inclusion. Young people, as a group, have advantages in making use of the internet; however, among the youth, these advantages vary according to where they live. Providing mandatory computer classes and sufficient digital practices for the youth who live in remote countries is necessary to improve their digital literacy. Furthermore, community care centres and schools for the aged may provide internet access and use opportunities for the elderly. While access to the internet is a private matter, flexible strategic policies should provide people with different entries to internet access or use. If they are willing to start, people could immediately take action.

Second, the disparities in online activity preferences are based on socioeconomic status and age. Compared with youth aged below 24 years (who are typically regarded as digital natives), middle-aged adults place more importance on the working activity in the 2018 panel. There are no changes in the studying, entertainment, or socialising online activities. Socioeconomic backgrounds determine the preferences for capital-enhancing activities or recreational activities.

Therefore, abundant open accessible resources are helpful for the people who are at a lower status but still willing to use capital-enhancing activities. For example, online open universities and public libraries may offer various kinds of technical courses to people at different levels to improve online users’ digital literacy.

Third, the transformation outcomes concern the integrated measures of digital capital to other social capitals. The correlation analysis reveals a positive association between capital-enhancing activities and socioeconomic statuses over 10 years. In the analysis of the cohort effect over 10 years, the same cohort deepened its ICT usage, especially the digital natives and digital immigrants, which showed digital inclusion. The outcome for individual internet usage shows that the higher the social status, the greater the preference for capital-enhancing activities; however, the weights of individuals with relatively low socioeconomic status decreased between the panels. Although no concrete evidence suggests that online internet usage preferences in the 2010 panel—especially the capital-enhancing activities—led to an increase in socioeconomic statuses in the 2018 panel, the factors for the ICT usage preferences in the 2010 panel and socioeconomic statuses in the 2018 panel are co-dependent. Therefore, time, as an integrated context in politics, economics, society, and culture, implicitly reflects the development of digital governance, digital economy, and digital society; accordingly, the participants have changed their recognition of technologies.

As part of the digital inclusion process, digital platforms and online users are competing for the use of data; yet, online users typically remain in an inferior position. Based on the multi-sided characteristic of the digital market, online users exchange their data for the digital platforms’ products or services. Greater data collection from digital platforms can provide more online users with more individualised and customised products or services. However, due to the strong competition among digital platforms, the platforms must collect more consumer data, and process or use them for a variety of purposes to gain more competitive advantages [53,54]. Consumer data are regarded as a production input, strategic asset used to maintain dominance among rivals, and valuable commodity to be sold to other businesses; therefore, consumer data are recognised as a critical asset [54]. Meanwhile, with the digital platforms’ high demand for more data collection, online users face information asymmetry because they have difficulty in understanding whether their data have been collected, and who has collected it. Digital platforms can even covertly collect user data without user consent [53,55]. Therefore, since 2012, China has established a private information protection system as a legal, justifiable, and informed consent system, which is embodied in the Civil Code and Personal Information Protection Law of the People’s Republic of China [56]. Furthermore, while the relevant government bureaus promulgate regulations and norms, the digital platforms formulate their own privacy protection policies to express the relevant rights, obligations, and responsibilities regarding users’ data protection [55]. However, regarding online users’ informed consent, three situations arise that concern private data protection and risk of consent to data exposure: unwitting consent, coerced consent, and incapacitated consent. Unwitting consent refers to the complicated privacy protection policies supplied by digital platforms; online users may skim through these policies because of differences in cognitive abilities and indifferent states of mind [56]. Meanwhile, a lack of alternatives may also force online users to consent to privacy protection policies. Giant digital platforms make use of their dominant market power to limit their rivals, erode consumer welfare, and force online consumers to consent to their data policies to receive online products or services [56,57]. Generally, digital platforms first develop and then regulate their data protection policies. In the initial stage, China adopted an inclusive and prudent supervision method to develop internet companies. At present, due to the problems between online users and digital platforms, China is gradually adopting strengthened supervision and governance to protect the rights of online users and retain competition in the digital market [57]. Accordingly, online users and digital platforms may have a clearer understanding of data protection and data collection through clear and readable privacy protection policies, multiple options for the platforms to obtain consent for data collection, and diverse digital platform alternatives for related products or services.

ICT use as a digital human capital aimed towards capital-enhancing activities has helped online users to increase their socioeconomic status; however, it has also exacerbated existing social inequalities. This study reveals the disparities in the digital inclusion process through the acceptance time, online activity preferences, and outcomes that lead to comparative inequality, as the individuals who receive greater or earlier benefits from ICT use benefit from the condition of relative inequality [9]. Meanwhile, absolute inequalities still exist. This study focuses on individuals who already possess the necessary equipment and skills for ICT access and use. People with low socioeconomic status participate less in both online and offline activities [12]. Therefore, digital exclusion is a clear threat to the community and economy because it is likely to enlarge other social, economic, cultural, ethnic, and gender disparities [9,17,21]. Closing the digital gap should be compatible with simultaneously reducing other social inequalities. Meanwhile, sufficient state governance can help the digitally excluded to become digitally included. For example, as older adults may easily be excluded from digital life, multi-level changes aimed at them could first include introducing smartphones that are specifically tailored towards older adults through sufficient screen sizes, font sizes, volumes, battery voltage, and ease of use. Second, websites or apps could design special pages that feature simplified procedures and easy-to-read information for older adults. Third, older adults could be provided with technical education from their relatives, senior universities, or communal institutions [58].

## 6. Conclusions

Therefore, Hypothesis 1 (depth of internet use) is almost supported, while Hypotheses 1B and 1D (income) are statistically insignificant. Hypothesis 2A (width of internet use) is partially supported as individuals with a high educational background prefer studying and working online, while individuals with high socioeconomic status prefer to work online; however, the selection of studying activity is not impacted by status. Hypothesis 2B is rejected because there are no clear cohort preferences regarding educational background and socioeconomic status on the entertainment and social activity preferences. Therefore, through these 10 years of digital inclusion in China, we observed a downward spread of internet use based on socioeconomic status and a relatively clear capital-enhancing activities’ preference for people in upper socioeconomic statuses.

The study contributions are as follows. First, this study responds to Gallardo et al. [21] by selecting a national representative sample to focus on specific netizen groups and reveal the dynamic digital inclusion process in China. The time effect is explored at the macro level to present a national condition of the online activity preferences, and the cohort effect is explored at the micro level to show the online behavioural changes of a specific cohort over 10 years. Second, this study confirms that individuals with different socioeconomic statuses show different preferences towards capital-enhancing and recreational activities, which supports Hargittai & Hinnant [19]. Third, policymakers, practitioners, and others can use the findings to help reduce the second gap of the digital divide and enhance digital inclusion.

Furthermore, digital inclusion and digital exclusion are two sides of the same coin. Strategic policies should first reduce the burdens of users related to mobile coverage and make internet accessibility affordable; second, offer accessible opportunities and abundant resources to people to enhance their digital literacy; third, supply sufficient guidance to online users and provide adequate surveillance for digital platforms. For example, youth can be guided on internet usage in two main ways. On the one hand, young people can resort to information classes at school and different digital creative contests to enhance their digital use capability, which will help them consciously make use of capital-enhancing activities; on the other hand, their overuse of online recreational activities should be restricted to avoid digital addiction.

This study has the following limitations. First, we used individual data to measure the importance of different online use behaviours, which may have data biases due to the self-reported nature. Second, as a result of adjusting the questionnaires among the two panels, the internet usage frequency lacked similarities in the panels. Third, this study did not include a selection of business behaviours, so it used the evaluations of the importance of the four online activities to reveal individuals’ internet use behaviours.

Future research could focus on several investigations: first, to define what kind of internet use behaviour belongs to capital-enhancing activities; second, based on the micro-level data, to explore the effects of diverse internet use behaviours on individuals; third, to explain the theoretical reasons for the effects of capital-enhancing activities on individuals.

## Figures and Tables

**Table 1:** Descriptive statistical analysis of the variables.

Panel	2010				2018				Sig.
Variable	Obs	Mean	Std. Dev.	Range	Obs	Mean	Std. Dev.	Range	
Time Effect									
Internet time	6278	2.131	2.315	[0, 24]	17,505	13.540	12.831	[0, 168]	***
Age range									
less than 24 years old	(base)				(base)				***
25–31 years old	6278	0.265	0.441	[0, 1]	17,505	0.230	0.421	[0, 1]	***
32–49 years old	6278	0.288	0.453	[0, 1]	17,505	0.358	0.480	[0, 1]	***
more than 50 years old	6278	0.067	0.251	[0, 1]	17,505	0.174	0.379	[0, 1]	***
Gender	6278	0.451	0.498	[0, 1]	17,505	0.480	0.500	[0, 1]	***
Urban	6278	0.691	0.462	[0, 1]	17,259	0.585	0.493	[0, 1]	***
Education									
less than elementary school	(base)				(base)				*
middle school	6278	0.337	0.473	[0, 1]	17,504	0.334	0.472	[0, 1]	***
high school	6278	0.304	0.460	[0, 1]	17,504	0.224	0.417	[0, 1]	***
beyond high school	6278	0.269	0.443	[0, 1]	17,504	0.196	0.397	[0, 1]	***
Income status									
low	(base)				(base)				
lower middle	5209	0.245	0.430	[0, 1]	14,608	0.195	0.396	[0, 1]	**
middle	5209	0.442	0.497	[0, 1]	14,608	0.524	0.499	[0, 1]	***
high	5209	0.067	0.251	[0, 1]	14,608	0.180	0.384	[0, 1]	
Social status									
low	(base)				(base)				
lower middle	6263	0.190	0.393	[0, 1]	15,955	0.176	0.381	[0, 1]	
middle	6263	0.563	0.496	[0, 1]	15,955	0.518	0.500	[0, 1]	***
high	6263	0.120	0.325	[0, 1]	15,955	0.215	0.411	[0, 1]	
Online activity evaluation									
Studying	6272	3.384	1.172	[1, 5]	17,505	2.668	1.633	[1, 5]	***
Working	6260	3.201	1.403	[1, 5]	17,505	2.171	1.694	[1, 5]	***
Entertainment	6274	3.183	1.190	[1, 5]	17,504	3.177	1.349	[1, 5]	***
Social activity	6269	3.179	1.225	[1, 5]	17,503	3.627	1.385	[1, 5]	***
ISEI	3369	45.927	16.411	[19, 88]	12,354	39.741	15.451	[19, 90]	***
**Cohort effect**									
Age cohort (Birth year)									
1987–1994	(base)				(base)				NA
1979–1986	3042	0.253	0.435	[0, 1]	3042	0.253	0.435	[0, 1]	NA
1960–1978	3042	0.301	0.459	[0, 1]	3042	0.301	0.459	[0, 1]	NA
before 1960	3042	0.066	0.248	[0, 1]	3042	0.066	0.248	[0, 1]	NA
Gender	3042	0.433	0.496	[0, 1]	3042	0.433	0.496	[0, 1]	NA
Urban	3042	0.655	0.476	[0, 1]	3003	0.753	0.431	[0, 1]	***
Education									
less than middle school	(base)				(base)				***
high school	3042	0.311	0.463	[0, 1]	3042	0.260	0.439	[0, 1]	***
beyond high school	3042	0.253	0.435	[0, 1]	3042	0.443	0.497	[0, 1]	***
Income status									
low	(base)				(base)				***
lower middle	2520	0.250	0.433	[0, 1]	2996	0.189	0.392	[0, 1]	***
middle	2520	0.447	0.497	[0, 1]	2996	0.570	0.495	[0, 1]	***
high	2520	0.061	0.239	[0, 1]	2996	0.161	0.367	[0, 1]	***
Social status									
low	(base)				(base)				***
lower middle	3032	0.188	0.391	[0, 1]	3037	0.185	0.389	[0, 1]	***
middle	3032	0.573	0.495	[0, 1]	3037	0.558	0.497	[0, 1]	***
high	3032	0.118	0.323	[0, 1]	3037	0.184	0.387	[0, 1]	***
Online activity evaluation									
Studying	3040	3.402	1.159	[1, 5]	3042	3.141	1.653	[1, 5]	***
Working	3036	2.355	1.145	[1, 4]	3042	2.483	1.367	[1, 4]	***
Entertainment	3041	3.185	1.175	[1, 5]	3042	3.441	1.291	[1, 5]	***
Social activity	3040	3.205	1.220	[1, 5]	3042	4.005	1.222	[1, 5]	***

Whether a variable shows a difference among the 2010 and 2018 panels was checked by the chi-square test, and marked with *** *p* < 0.01, ** *p* < 0.05, * *p* < 0.10. Variables of age cohorts and gender in the cohort effect are not available.

**Table 2:** Results of the multiple regression model predicting the frequency of internet use in the 2010 and 2018 panels.

Panel		
	2010	2018
Age range		
25–31 years old	−0.215 (0.126)	−2.838 *** (0.374)
32–49 years old	−0.707 *** (0.129)	−7.007 *** (0.472)
more than 50 years old	−0.593 *** (0.125)	−8.003 *** (0.567)
Female	−0.174 *** (0.051)	0.423 (0.266)
Urban	0.397 *** (0.088)	0.927 *** (0.283)
Education		
middle school	0.096 (0.088)	2.006 *** (0.466)
high school	0.524 *** (0.099)	3.192 *** (0.581)
beyond high school	0.962 *** (0.211)	2.759 *** (0.572)
Income status		
lower middle	−0.042 (0.098)	0.409 (0.354)
middle	0.093 (0.117)	0.049 (0.344)
high	0.194 (0.127)	−0.353 (0.462)
Social status		
lower middle	−0.299 ** (0.132)	0.253 (0.494)
middle	−0.287 ** (0.104)	−0.125 (0.383)
high	−0.457 *** (0.137)	−1.030 ** (0.393)
Constant	2.128 *** (0.191)	16.502 *** (0.854)
Observations	5208	14,367
R-squared	0.044	0.067

Note. Robust standard errors in parentheses, *** *p* < 0.01, ** *p* < 0.05, * *p* < 0.10. Consequently, reference categories are less than 24 years old, male, rural-living, less than elementary school, low income status, and low social status.

**Table 3:** Partial results from the generalised partial proportional odds model of the ISEI scores predicting the importance of online activities in the 2010 and 2018 panels (robustness test).

Panel	2010				2018			
Evaluations	1	2	3	4	1	2	3	4
**Studying activity**								
Lower middle	−0.080	−0.080	−0.080	−0.080	−0.115 **	−0.115 **	−0.115 **	−0.115 **
	(0.063)	(0.063)	(0.063)	(0.063)	(0.055)	(0.055)	(0.055)	(0.055)
Middle	0.120 *	0.120 *	0.120 *	0.120 *	0.315 ***	0.315 ***	0.315 ***	0.315 ***
	(0.066)	(0.066)	(0.066)	(0.066)	(0.092)	(0.092)	(0.092)	(0.092)
High	1.234 ***	0.709 ***	0.608 ***	0.516 ***	1.522 ***	1.450 ***	1.276 ***	1.032 ***
	(0.144)	(0.112)	(0.065)	(0.074)	(0.102)	(0.093)	(0.077)	(0.060)
Observations	6272	6272	6272	6272	17,259	17,259	17,259	17,259
**Working activity**								
Lower middle	0.255 ***	0.255 ***	0.255 ***	0.255 ***	0.858 ***	0.830 ***	0.776 ***	0.647 ***
	(0.054)	(0.054)	(0.054)	(0.054)	(0.063)	(0.062)	(0.064)	(0.076)
Middle	0.812 ***	0.773 ***	0.668 ***	0.606 ***	1.800 ***	1.782 ***	1.742 ***	1.611 ***
	(0.113)	(0.071)	(0.068)	(0.072)	(0.072)	(0.075)	(0.073)	(0.086)
High	1.621 ***	1.385 ***	1.277 ***	1.088 ***	3.031 ***	3.013 ***	2.850 ***	2.497 ***
	(0.123)	(0.096)	(0.100)	(0.084)	(0.087)	(0.087)	(0.073)	(0.073)
Observations	6260	6260	6260	6260	17,259	17,259	17,259	17,259
**Entertainment activity**								
Lower middle	0.039	0.039	0.039	0.039	0.360 ***	0.360 ***	0.360 ***	0.360 ***
	(0.069)	(0.069)	(0.069)	(0.069)	(0.078)	(0.078)	(0.078)	(0.078)
Middle	0.033	0.033	0.033	0.033	0.455 ***	0.455 ***	0.455 ***	0.455 ***
	(0.082)	(0.082)	(0.082)	(0.082)	(0.066)	(0.066)	(0.066)	(0.066)
High	−0.244 ***	−0.244 ***	−0.244 ***	−0.244 ***	0.595 ***	0.467 ***	0.420 ***	0.406 ***
	(0.057)	(0.057)	(0.057)	(0.057)	(0.093)	(0.071)	(0.063)	(0.069)
Observations	6274	6274	6274	6274	17,258	17,258	17,258	17,258
**Social activity**								
Lower middle	0.147 ***	0.147 ***	0.147 ***	0.147 ***	0.489 ***	0.478 ***	0.342 ***	0.376 ***
	(0.050)	(0.050)	(0.050)	(0.050)	(0.084)	(0.075)	(0.055)	(0.047)
Middle	0.245 *	0.080	−0.014	0.018	0.842 ***	0.881 ***	0.684 ***	0.686 ***
	(0.126)	(0.105)	(0.101)	(0.123)	(0.057)	(0.068)	(0.064)	(0.058)
High	0.255 **	0.084	−0.017	−0.197 **	1.198 ***	1.105 ***	0.924 ***	0.815 ***
	(0.114)	(0.097)	(0.083)	(0.096)	(0.085)	(0.061)	(0.060)	(0.050)
Observations	6269	6269	6269	6269	17,257	17,257	17,257	17,257

Note. Robust standard errors in parentheses, *** *p* < 0.01, ** *p* < 0.05, * *p* < 0.10. Reference category is low ISEI. The four panels of internet activity evaluation included the 1st panel (category 1 versus categories 2, 3, 4 and 5); 2nd panel (categories 1 and 2 versus categories 3, 4, and 5); 3rd panel (categories 1, 2 and 3 versus categories 4 and 5); and 4th panel (categories 1, 2, 3 and 4 versus category 5). The complete table is in Supplemental Material Table S2.

**Table 4:** Correlation analysis between the importance of the online activities in the 2010 panel and the ISEI scores in the 2018 panel.

	Online Activities in the 2010 Panel			
	Studying	Working	Social Activity	Entertainment
**ISEI in the 2018 panel**	0.1217 ***	0.1669 ***	0.0706 ***	−0.0120

Note. Significance has been checked and marked with *** *p* < 0.01, ** *p* < 0.05, * *p* < 0.10.

## Data Availability

The data presented in this study are available in the China Family Panel Studies, Peking University Open Research Data at doi:10.18170/DVN/45LCSO, accessed on 5 April 2021.

## Supplementary Materials

The following supporting information can be downloaded at: https://www.mdpi.com/article/10.3390/ijerph191911937/s1, Table S1: Results of the generalised partial proportional odds model predicting the importance of online activities in the 2010 and 2018 panels; Table S2: Results of the generalised partial proportional odds model of ISEI predicting the importance of online activities in the 2010 and 2018 panels (robustness test); Table S3: Results of the generalised partial proportional odds model of cohorts predicting the importance of online activities in the 2010 and 2018 panels.

## Appendix A

**Table A1:** Independence test between explanatory and explained variables using a chi-square test.

Time Effect									
Panel			Age Range	Gender	Urban	Education	Income Status	Social Status	ISEI
2010	Internet time		***	***	***	***	***	***	***
	Online activity	Study	***	***	**	***	***	***	***
		Work	***		***	***	***	***	***
		Entertainment	***	*	***	***	***	***	
		Social activity	***		***	***	***	***	**
2018	Internet time		***	***	***	***	***	***	
	Online activity	Study	***	***	***	***	***	***	***
		Work	***	***	***	***	***	***	***
		Entertainment	***	***	***	***	***	***	***
		Social activity	***	***	***	***	***	***	***
**Cohort Effect**									
**Panel**			**Age Cohort**	**Gender**	**Urban**	**Education**	**Income Status**	**Social Status**	
2010	Internet time		***	**	***	***			
	Online activity	Study	***		***	***	***	***	
		Work	***		***	***	***	***	
		Entertainment	***		***		**	***	
		Social activity	***		***	***	**	***	
2018	Internet time		***	***		***			
	Online activity	Study	***	*	***	***	***	***	
		Work	***	***	***	***	***	***	
		Entertainment	***	***	*	***	***	***	
		Social activity	***	***		***	***	***	

Results are marked with *** *p* < 0.01, ** *p* < 0.05, * *p* < 0.10.
