# Supplementary material for: Disparities in Online Use Behaviours and Chinese Digital Inclusion: A 10-Year Comparison
Source: Int J Environ Res Public Health. 2022 Sep 21;19(19):11937. doi: 10.3390/ijerph191911937 (PMC9565673; doi:10.3390/ijerph191911937)
Supplement: Supplementary file 1 [file ijerph-19-11937-s001.zip › ijerph-1887994-supplementary.pdf]

# Disparities in Online Use Behaviours and Chinese Digital Inclusion: A 10-year Comparison

## Supplementary Material List

Table S1. Results of the generalised partial proportional odds model predicting the importance of the online activities in the 2010 and 2018 panels. page 2

Table S2. Results of the generalised partial proportional odds model of ISEI predicting the importance of the online activities in the 2010 and 2018 panels (robustness test). page 6

Table S3. Results of the generalised partial proportional odds model of cohorts predicting the importance of the online activities in the 2010 and 2018 panels. page 9

**Table S1.** Results of the generalised partial proportional odds model predicting the importance of the online activities in the 2010 and 2018 panels.

| **Panel** | **2010** | | | | **2018** | | | |
|---|---|---|---|---|---|---|---|---|
| **Evaluation** | **1** | **2** | **3** | **4** | **1** | **2** | **3** | **4** |
| | | | | **Studying activity** | | | | |
| Age range (ref. less than 24 years old) | | | | | | | | |
| 25-31 years old | -0.146 | -0.184*** | -0.188*** | 0.027 | -1.323*** | -1.029*** | -0.203*** | 0.315*** |
| | (0.116) | (0.072) | (0.072) | (0.093) | (0.060) | (0.050) | (0.048) | (0.062) |
| 32-49 years old | -0.498*** | -0.322*** | -0.139 | 0.162 | -1.588*** | -1.279*** | -0.379*** | 0.239*** |
| | (0.104) | (0.076) | (0.100) | (0.112) | (0.057) | (0.049) | (0.041) | (0.060) |
| more than 50 years old | -1.399*** | -0.812*** | -0.408*** | -0.040 | -2.202*** | -1.886*** | -0.914*** | -0.370*** |
| | (0.211) | (0.146) | (0.128) | (0.154) | (0.084) | (0.080) | (0.068) | (0.094) |
| Female (ref. male) | 0.150*** | 0.150*** | 0.150*** | 0.150*** | -0.096*** | -0.063* | -0.029 | -0.116*** |
| | (0.047) | (0.047) | (0.047) | (0.047) | (0.032) | (0.033) | (0.035) | (0.035) |
| Urban (ref. rural-living) | 0.017 | 0.017 | 0.017 | 0.017 | 0.178*** | 0.157*** | 0.051 | 0.043 |
| | (0.057) | (0.057) | (0.057) | (0.057) | (0.047) | (0.046) | (0.037) | (0.046) |
| Education (ref. less than elementary school) | | | | | | | | |
| middle school | 0.608*** | 0.514*** | 0.178* | -0.151 | 0.464*** | 0.458*** | 0.353*** | 0.214*** |
| | (0.147) | (0.103) | (0.098) | (0.102) | (0.094) | (0.092) | (0.081) | (0.077) |
| high school | 1.335*** | 0.930*** | 0.556*** | 0.062 | 1.218*** | 1.192*** | 1.022*** | 0.718*** |
| | (0.144) | (0.100) | (0.081) | (0.118) | (0.092) | (0.097) | (0.079) | (0.065) |
| beyond high school | 2.070*** | 1.504*** | 0.886*** | 0.285** | 2.385*** | 2.320*** | 1.969*** | 1.458*** |
| | (0.214) | (0.134) | (0.091) | (0.127) | (0.140) | (0.135) | (0.100) | (0.063) |
| Income status (ref. low) | | | | | | | | |
| lower middle | -0.269*** | -0.269*** | -0.269*** | -0.269*** | -0.259*** | -0.259*** | -0.259*** | -0.259*** |
| | (0.062) | (0.062) | (0.062) | (0.062) | (0.071) | (0.071) | (0.071) | (0.071) |
| middle | -0.246*** | -0.246*** | -0.246*** | -0.246*** | -0.161*** | -0.161*** | -0.161*** | -0.161*** |
| | (0.056) | (0.056) | (0.056) | (0.056) | (0.045) | (0.045) | (0.045) | (0.045) |
| high | -0.244** | -0.244** | -0.244** | -0.244** | -0.250*** | -0.219*** | -0.047 | -0.006 |
| | (0.106) | (0.106) | (0.106) | (0.106) | (0.048) | (0.048) | (0.058) | (0.057) |
| Social status (ref. low) | | | | | | | | |
| lower middle | 0.532*** | 0.114 | 0.167 | -0.287* | -0.204** | -0.246*** | -0.321*** | -0.419*** |
| | (0.126) | (0.096) | (0.108) | (0.157) | (0.095) | (0.092) | (0.086) | (0.108) |
| middle | 0.782*** | 0.490*** | 0.372*** | -0.053 | -0.038 | -0.074 | -0.204*** | -0.337*** |
| | (0.131) | (0.097) | (0.120) | (0.155) | (0.048) | (0.051) | (0.057) | (0.065) |
| high | 1.054*** | 0.843*** | 0.840*** | 0.296** | 0.002 | 0.002 | 0.002 | 0.002 |
| | (0.221) | (0.116) | (0.160) | (0.144) | (0.058) | (0.058) | (0.058) | (0.058) |
| Constant | 1.173*** | 0.434*** | -0.673*** | -1.428*** | 0.868*** | 0.461*** | -0.812*** | -1.622*** |
| | (0.192) | (0.140) | (0.154) | (0.101) | (0.072) | (0.063) | (0.062) | (0.056) |
| Observations | 6,272 | 6,272 | 6,272 | 6,272 | 17,259 | 17,259 | 17,259 | 17,259 |
| Pseudo R square | 0.0280 | 0.0280 | 0.0280 | 0.0280 | 0.106 | 0.106 | 0.106 | 0.106 |
| | | | | **Working activity** | | | | |
| Age range (ref. less than 24 years old) | | | | | | | | |

| | | | | | | | | |
|---|---|---|---|---|---|---|---|---|
| 25-31 years old | 0.264*** | 0.158** | 0.064 | 0.005 | 0.851*** | 0.851*** | 0.851*** | 0.851*** |
| | (0.092) | (0.080) | (0.050) | (0.049) | (0.084) | (0.084) | (0.084) | (0.084) |
| 32-49 years old | -0.023 | -0.023 | -0.023 | -0.023 | 0.764*** | 0.764*** | 0.764*** | 0.764*** |
| | (0.063) | (0.063) | (0.063) | (0.063) | (0.103) | (0.103) | (0.103) | (0.103) |
| more than 50 years old | -1.185*** | -0.963*** | -0.699*** | -0.681*** | -0.380*** | -0.380*** | -0.380*** | -0.380*** |
| | (0.124) | (0.115) | (0.140) | (0.120) | (0.139) | (0.139) | (0.139) | (0.139) |
| Female (ref. male) | 0.094*** | 0.094*** | 0.094*** | 0.094*** | -0.375*** | -0.310*** | -0.234*** | -0.230*** |
| | (0.035) | (0.035) | (0.035) | (0.035) | (0.030) | (0.031) | (0.037) | (0.040) |
| Urban (ref. rural-living) | 0.060 | 0.060 | 0.060 | 0.060 | 0.250*** | 0.250*** | 0.250*** | 0.250*** |
| | (0.056) | (0.056) | (0.056) | (0.056) | (0.045) | (0.045) | (0.045) | (0.045) |
| Education (ref. less than elementary school) | | | | | | | | |
| middle school | 0.054 | 0.054 | 0.054 | 0.054 | 0.868*** | 0.868*** | 0.868*** | 0.868*** |
| | (0.090) | (0.090) | (0.090) | (0.090) | (0.102) | (0.102) | (0.102) | (0.102) |
| high school | 0.557*** | 0.524*** | 0.389*** | 0.295*** | 1.546*** | 1.566*** | 1.571*** | 1.515*** |
| | (0.084) | (0.085) | (0.082) | (0.087) | (0.093) | (0.093) | (0.089) | (0.086) |
| beyond high school | 1.122*** | 1.104*** | 0.978*** | 0.847*** | 2.978*** | 3.011*** | 2.889*** | 2.601*** |
| | (0.118) | (0.118) | (0.102) | (0.089) | (0.112) | (0.106) | (0.100) | (0.085) |
| Income status (ref. low) | | | | | | | | |
| lower middle | 0.264*** | 0.264*** | 0.264*** | 0.264*** | 1.391*** | 1.352*** | 1.246*** | 1.066*** |
| | (0.081) | (0.081) | (0.081) | (0.081) | (0.092) | (0.088) | (0.096) | (0.093) |
| middle | 0.605*** | 0.444*** | 0.449*** | 0.450*** | 1.621*** | 1.624*** | 1.546*** | 1.316*** |
| | (0.095) | (0.071) | (0.075) | (0.067) | (0.103) | (0.102) | (0.103) | (0.095) |
| high | 1.101*** | 0.704*** | 0.608*** | 0.581*** | 1.580*** | 1.592*** | 1.565*** | 1.360*** |
| | (0.206) | (0.163) | (0.135) | (0.107) | (0.109) | (0.111) | (0.116) | (0.128) |
| Social status (ref. low) | | | | | | | | |
| lower middle | 0.164 | -0.000 | -0.147 | -0.539*** | -0.134* | -0.193*** | -0.305*** | -0.340*** |
| | (0.138) | (0.102) | (0.112) | (0.155) | (0.074) | (0.072) | (0.076) | (0.082) |
| middle | 0.311** | 0.205** | -0.012 | -0.431*** | -0.177*** | -0.214*** | -0.288*** | -0.376*** |
| | (0.133) | (0.099) | (0.108) | (0.150) | (0.068) | (0.067) | (0.064) | (0.076) |
| high | 0.608*** | 0.519*** | 0.382*** | -0.142 | -0.143** | -0.143** | -0.143** | -0.143** |
| | (0.155) | (0.126) | (0.135) | (0.182) | (0.070) | (0.070) | (0.070) | (0.070) |
| Constant | 0.503*** | -0.104 | -0.868*** | -1.516*** | -3.571*** | -3.676*** | -3.932*** | -4.020*** |
| | (0.160) | (0.118) | (0.121) | (0.152) | (0.090) | (0.097) | (0.098) | (0.104) |
| Observations | 6,260 | 6,260 | 6,260 | 6,260 | 17,259 | 17,259 | 17,259 | 17,259 |
| Pseudo R square | 0.0349 | 0.0349 | 0.0349 | 0.0349 | 0.187 | 0.187 | 0.187 | 0.187 |
| **Entertainment activity** | | | | | | | | |
| Age range (ref. less than 24 years old) | | | | | | | | |
| 25-31 years old | -0.495*** | -0.239*** | -0.234*** | -0.025 | -0.742*** | -0.262*** | 0.085* | 0.506*** |
| | (0.106) | (0.073) | (0.078) | (0.094) | (0.092) | (0.059) | (0.047) | (0.064) |
| 32-49 years old | -1.049*** | -0.770*** | -0.556*** | -0.215** | -1.451*** | -0.852*** | -0.440*** | 0.029 |
| | (0.107) | (0.092) | (0.074) | (0.087) | (0.085) | (0.056) | (0.054) | (0.061) |
| more than 50 years old | -1.609*** | -1.162*** | -0.864*** | -0.173* | -1.915*** | -1.152*** | -0.515*** | -0.076 |
| | (0.152) | (0.120) | (0.111) | (0.105) | (0.114) | (0.074) | (0.063) | (0.080) |
| Female (ref. male) | -0.032 | 0.024 | -0.126** | -0.066 | 0.105*** | 0.128*** | 0.153*** | 0.050 |

| | | | | | | | | |
|---|---|---|---|---|---|---|---|---|
| | (0.110) | (0.046) | (0.055) | (0.065) | (0.029) | (0.030) | (0.034) | (0.041) |
| Urban (ref. rural-living) | 0.540*** | 0.468*** | 0.473*** | 0.287*** | 0.180*** | 0.180*** | 0.180*** | 0.180*** |
| | (0.178) | (0.121) | (0.091) | (0.099) | (0.066) | (0.066) | (0.066) | (0.066) |
| Education (ref. less than elementary school) | | | | | | | | |
| middle school | 0.167* | 0.167* | 0.167* | 0.167* | 0.442*** | 0.399*** | 0.310*** | 0.190*** |
| | (0.091) | (0.091) | (0.091) | (0.091) | (0.086) | (0.066) | (0.052) | (0.057) |
| high school | 0.285*** | 0.285*** | 0.285*** | 0.285*** | 0.732*** | 0.549*** | 0.433*** | 0.267*** |
| | (0.097) | (0.097) | (0.097) | (0.097) | (0.097) | (0.069) | (0.062) | (0.074) |
| beyond high school | 0.168* | 0.168* | 0.168* | 0.168* | 0.947*** | 0.670*** | 0.527*** | 0.302*** |
| | (0.096) | (0.096) | (0.096) | (0.096) | (0.094) | (0.070) | (0.075) | (0.089) |
| Income status (ref. low) | | | | | | | | |
| lower middle | 0.081 | 0.081 | 0.081 | 0.081 | 0.164** | 0.083 | 0.165** | 0.197** |
| | (0.117) | (0.117) | (0.117) | (0.117) | (0.068) | (0.058) | (0.066) | (0.081) |
| middle | 0.220*** | 0.220*** | 0.220*** | 0.220*** | 0.290*** | 0.290*** | 0.290*** | 0.290*** |
| | (0.085) | (0.085) | (0.085) | (0.085) | (0.063) | (0.063) | (0.063) | (0.063) |
| high | 0.466*** | 0.466*** | 0.466*** | 0.466*** | 0.029 | 0.110* | 0.393*** | 0.511*** |
| | (0.158) | (0.158) | (0.158) | (0.158) | (0.067) | (0.058) | (0.071) | (0.076) |
| Social status (ref. low) | | | | | | | | |
| lower middle | 0.197** | 0.020 | 0.043 | -0.384*** | 0.397*** | 0.149*** | -0.108 | -0.188** |
| | (0.092) | (0.136) | (0.095) | (0.126) | (0.107) | (0.047) | (0.067) | (0.085) |
| middle | 0.406*** | 0.207 | 0.102 | -0.393*** | 0.445*** | 0.322*** | -0.001 | -0.174** |
| | (0.154) | (0.138) | (0.119) | (0.145) | (0.067) | (0.052) | (0.067) | (0.077) |
| high | 0.370*** | 0.169 | 0.300* | -0.288 | 0.230*** | 0.207*** | 0.082 | -0.041 |
| | (0.132) | (0.170) | (0.171) | (0.196) | (0.079) | (0.058) | (0.079) | (0.077) |
| Constant | 1.810*** | 0.643*** | -0.875*** | -1.732*** | 1.700*** | 0.635*** | -0.896*** | -1.810*** |
| | (0.258) | (0.169) | (0.163) | (0.187) | (0.142) | (0.120) | (0.106) | (0.096) |
| Observations | 6,274 | 6,274 | 6,274 | 6,274 | 17,258 | 17,258 | 17,258 | 17,258 |
| Pseudo R square | 0.0177 | 0.0177 | 0.0177 | 0.0177 | 0.0339 | 0.0339 | 0.0339 | 0.0339 |
| **Social activity** | | | | | | | | |
| Age range (ref. less than 24 years old) | | | | | | | | |
| 25-31 years old | -0.292*** | -0.292*** | -0.292*** | -0.292*** | -0.300*** | 0.083 | 0.234*** | 0.603*** |
| | (0.064) | (0.064) | (0.064) | (0.064) | (0.060) | (0.059) | (0.035) | (0.059) |
| 32-49 years old | -1.332*** | -1.217*** | -0.982*** | -0.869*** | -0.879*** | -0.537*** | -0.259*** | 0.206*** |
| | (0.097) | (0.085) | (0.066) | (0.120) | (0.061) | (0.050) | (0.048) | (0.060) |
| more than 50 years old | -2.262*** | -1.956*** | -1.516*** | -1.136*** | -1.609*** | -1.148*** | -0.800*** | -0.312*** |
| | (0.139) | (0.138) | (0.139) | (0.177) | (0.102) | (0.094) | (0.092) | (0.095) |
| Female (ref. male) | -0.069 | -0.069 | -0.069 | -0.069 | 0.317*** | 0.219*** | 0.104*** | 0.009 |
| | (0.042) | (0.042) | (0.042) | (0.042) | (0.043) | (0.049) | (0.039) | (0.039) |
| Urban (ref. rural-living) | 0.093 | 0.093 | 0.093 | 0.093 | 0.136*** | 0.136*** | 0.136*** | 0.136*** |
| | (0.072) | (0.072) | (0.072) | (0.072) | (0.037) | (0.037) | (0.037) | (0.037) |
| Education (ref. less than elementary school) | | | | | | | | |
| middle school | 0.605*** | 0.320*** | 0.300*** | 0.105 | 0.457*** | 0.511*** | 0.428*** | 0.360*** |
| | (0.131) | (0.115) | (0.099) | (0.110) | (0.089) | (0.076) | (0.066) | (0.059) |
| high school | 0.911*** | 0.403*** | 0.359*** | 0.165 | 0.944*** | 0.937*** | 0.798*** | 0.541*** |

| | | | | | | | | |
|---|---|---|---|---|---|---|---|---|
| | (0.130) | (0.112) | (0.093) | (0.125) | (0.085) | (0.065) | (0.056) | (0.076) |
| beyond high school | 0.870*** | 0.422*** | 0.293* | 0.085 | 1.491*** | 1.368*** | 1.110*** | 0.863*** |
| | (0.115) | (0.145) | (0.163) | (0.108) | (0.128) | (0.087) | (0.067) | (0.053) |
| Income status (ref. low) | | | | | | | | |
| lower middle | -0.021 | -0.021 | -0.021 | -0.021 | 0.339*** | 0.205*** | 0.197*** | 0.237*** |
| | (0.098) | (0.098) | (0.098) | (0.098) | (0.062) | (0.058) | (0.047) | (0.045) |
| middle | 0.012 | 0.012 | 0.012 | 0.012 | 0.381*** | 0.381*** | 0.381*** | 0.381*** |
| | (0.052) | (0.052) | (0.052) | (0.052) | (0.049) | (0.049) | (0.049) | (0.049) |
| high | 0.113 | 0.113 | 0.113 | 0.113 | 0.066 | 0.108 | 0.393*** | 0.481*** |
| | (0.072) | (0.072) | (0.072) | (0.072) | (0.080) | (0.082) | (0.058) | (0.078) |
| Social status (ref. low) | | | | | | | | |
| lower middle | 0.462*** | 0.078 | -0.070 | -0.449** | 0.580*** | 0.454*** | 0.209** | 0.035 |
| | (0.104) | (0.101) | (0.126) | (0.180) | (0.073) | (0.067) | (0.083) | (0.085) |
| middle | 0.688*** | 0.491*** | 0.207** | -0.293 | 0.676*** | 0.573*** | 0.250*** | 0.025 |
| | (0.101) | (0.090) | (0.098) | (0.199) | (0.080) | (0.071) | (0.064) | (0.063) |
| high | 0.881*** | 0.691*** | 0.603*** | 0.162 | 0.552*** | 0.566*** | 0.455*** | 0.231*** |
| | (0.140) | (0.100) | (0.122) | (0.154) | (0.086) | (0.085) | (0.088) | (0.063) |
| Constant | 1.365*** | 0.889*** | -0.330*** | -1.357*** | 1.176*** | 0.451*** | -0.667*** | -1.515*** |
| | (0.193) | (0.164) | (0.112) | (0.176) | (0.104) | (0.096) | (0.078) | (0.071) |
| Observations | 6,269 | 6,269 | 6,269 | 6,269 | 17,257 | 17,257 | 17,257 | 17,257 |
| Pseudo R square | 0.0379 | 0.0379 | 0.0379 | 0.0379 | 0.0505 | 0.0505 | 0.0505 | 0.0505 |

*Note* Robust standard errors in parentheses, *** p<0.01, ** p<0.05, * p<0.10. Four panels of internet activity evaluation included 1st panel (category 1 versus categories 2, 3, 4, and 5); 2nd panel (categories 1 and 2 versus categories 3, 4, and 5); 3rd panel (categories 1, 2, and 3 versus categories 4 and 5); and 4th panel (categories 1, 2, 3, and 4 versus category 5).

**Table S2.** Results of the generalised partial proportional odds model of ISEI predicting the importance of the online activities in the 2010 and 2018 panels (robustness test).

| Panel | 2010 | | | | 2018 | | | |
|---|---|---|---|---|---|---|---|---|
| Evaluations | 1 | 2 | 3 | 4 | 1 | 2 | 3 | 4 |
| | | | | **Studying activity** | | | | |
| Age range (ref. less than 24 years old) | | | | | | | | |
| 25-31 years old | -0.170*** | -0.170*** | -0.170*** | -0.170*** | -1.131*** | -0.852*** | -0.104** | 0.285*** |
| | (0.059) | (0.059) | (0.059) | (0.059) | (0.061) | (0.052) | (0.049) | (0.060) |
| 32-49 years old | -0.549*** | -0.347*** | -0.173* | -0.001 | -1.689*** | -1.386*** | -0.531*** | 0.030 |
| | (0.106) | (0.088) | (0.097) | (0.097) | (0.070) | (0.071) | (0.053) | (0.060) |
| more than 50 years old | -1.331*** | -0.781*** | -0.389*** | -0.141 | -2.301*** | -1.994*** | -1.055*** | -0.547*** |
| | (0.208) | (0.149) | (0.118) | (0.145) | (0.073) | (0.071) | (0.059) | (0.076) |
| Female (ref. male) | 0.189*** | 0.189*** | 0.189*** | 0.189*** | -0.162*** | -0.120*** | -0.070** | -0.147*** |
| | (0.047) | (0.047) | (0.047) | (0.047) | (0.032) | (0.032) | (0.035) | (0.036) |
| Urban (ref. rural-living) | 0.447*** | 0.234*** | 0.119** | 0.015 | 0.408*** | 0.387*** | 0.238*** | 0.156*** |
| | (0.122) | (0.066) | (0.057) | (0.102) | (0.050) | (0.047) | (0.036) | (0.046) |
| ISEI (ref. low) | | | | | | | | |
| lower middle | -0.080 | -0.080 | -0.080 | -0.080 | -0.115** | -0.115** | -0.115** | -0.115** |
| | (0.063) | (0.063) | (0.063) | (0.063) | (0.055) | (0.055) | (0.055) | (0.055) |
| middle | 0.120* | 0.120* | 0.120* | 0.120* | 0.315*** | 0.315*** | 0.315*** | 0.315*** |
| | (0.066) | (0.066) | (0.066) | (0.066) | (0.092) | (0.092) | (0.092) | (0.092) |
| high | 1.234*** | 0.709*** | 0.608*** | 0.516*** | 1.522*** | 1.450*** | 1.276*** | 1.032*** |
| | (0.144) | (0.112) | (0.065) | (0.074) | (0.102) | (0.093) | (0.077) | (0.060) |
| Constant | 2.237*** | 1.204*** | -0.197*** | -1.572*** | 1.155*** | 0.735*** | -0.603*** | -1.562*** |
| | (0.146) | (0.096) | (0.070) | (0.094) | (0.065) | (0.059) | (0.044) | (0.043) |
| Observations | 6,272 | 6,272 | 6,272 | 6,272 | 17,259 | 17,259 | 17,259 | 17,259 |
| | | | | **Working activity** | | | | |
| Age range (ref. less than 24 years old) | | | | | | | | |
| 25-31 years old | 0.387*** | 0.267*** | 0.165*** | 0.097* | 1.167*** | 1.167*** | 1.167*** | 1.167*** |
| | (0.086) | (0.082) | (0.052) | (0.059) | (0.083) | (0.083) | (0.083) | (0.083) |
| 32-49 years old | 0.034 | 0.034 | 0.034 | 0.034 | 0.849*** | 0.849*** | 0.849*** | 0.849*** |
| | (0.066) | (0.066) | (0.066) | (0.066) | (0.091) | (0.091) | (0.091) | (0.091) |
| more than 50 years old | -0.873*** | -0.703*** | -0.502*** | -0.553*** | -0.139 | -0.139 | -0.139 | -0.139 |
| | (0.125) | (0.112) | (0.153) | (0.112) | (0.123) | (0.123) | (0.123) | (0.123) |
| Female (ref. male) | 0.078** | 0.078** | 0.078** | 0.078** | -0.440*** | -0.379*** | -0.315*** | -0.301*** |
| | (0.032) | (0.032) | (0.032) | (0.032) | (0.032) | (0.035) | (0.042) | (0.042) |
| Urban (ref. rural-living) | 0.102* | 0.102* | 0.102* | 0.102* | 0.328*** | 0.328*** | 0.328*** | 0.328*** |
| | (0.055) | (0.055) | (0.055) | (0.055) | (0.058) | (0.058) | (0.058) | (0.058) |
| ISEI (ref. low) | | | | | | | | |
| lower middle | 0.255*** | 0.255*** | 0.255*** | 0.255*** | 0.858*** | 0.830*** | 0.776*** | 0.647*** |
| | (0.054) | (0.054) | (0.054) | (0.054) | (0.063) | (0.062) | (0.064) | (0.076) |
| middle | 0.812*** | 0.773*** | 0.668*** | 0.606*** | 1.800*** | 1.782*** | 1.742*** | 1.611*** |
| | (0.113) | (0.071) | (0.068) | (0.072) | (0.072) | (0.075) | (0.073) | (0.086) |

| | | | | | | | | |
|---|---|---|---|---|---|---|---|---|
| high | 1.621*** | 1.385*** | 1.277*** | 1.088*** | 3.031*** | 3.013*** | 2.850*** | 2.497*** |
| | (0.123) | (0.096) | (0.100) | (0.084) | (0.087) | (0.087) | (0.073) | (0.073) |
| Constant | 1.119*** | 0.378*** | -0.579*** | -1.623*** | -2.391*** | -2.499*** | -2.842*** | -3.125*** |
| | (0.075) | (0.064) | (0.063) | (0.070) | (0.054) | (0.050) | (0.046) | (0.056) |
| Observations | 6,260 | 6,260 | 6,260 | 6,260 | 17,259 | 17,259 | 17,259 | 17,259 |
| **Entertainment activity** | | | | | | | | |
| Age range (ref. less than 24 years old) | | | | | | | | |
| 25-31 years old | -0.355*** | -0.124** | -0.113* | 0.039 | -0.437*** | -0.080 | 0.157** | 0.491*** |
| | (0.109) | (0.062) | (0.059) | (0.076) | (0.104) | (0.082) | (0.063) | (0.071) |
| 32-49 years old | -0.849*** | -0.612*** | -0.401*** | -0.138 | -1.254*** | -0.724*** | -0.403*** | 0.013 |
| | (0.115) | (0.090) | (0.085) | (0.094) | (0.075) | (0.067) | (0.059) | (0.070) |
| more than 50 years old | -1.413*** | -1.013*** | -0.700*** | -0.092 | -1.674*** | -0.963*** | -0.381*** | 0.039 |
| | (0.178) | (0.130) | (0.119) | (0.119) | (0.068) | (0.057) | (0.064) | (0.075) |
| Female (ref. male) | -0.051 | 0.002 | -0.148*** | -0.081 | 0.100*** | 0.136*** | 0.165*** | 0.071 |
| | (0.108) | (0.049) | (0.055) | (0.070) | (0.026) | (0.031) | (0.039) | (0.047) |
| Urban (ref. rural-living) | 0.458*** | 0.458*** | 0.458*** | 0.458*** | 0.274*** | 0.206** | 0.225*** | 0.147** |
| | (0.100) | (0.100) | (0.100) | (0.100) | (0.095) | (0.084) | (0.063) | (0.063) |
| ISEI (ref. low) | | | | | | | | |
| lower middle | 0.039 | 0.039 | 0.039 | 0.039 | 0.360*** | 0.360*** | 0.360*** | 0.360*** |
| | (0.069) | (0.069) | (0.069) | (0.069) | (0.078) | (0.078) | (0.078) | (0.078) |
| middle | 0.033 | 0.033 | 0.033 | 0.033 | 0.455*** | 0.455*** | 0.455*** | 0.455*** |
| | (0.082) | (0.082) | (0.082) | (0.082) | (0.066) | (0.066) | (0.066) | (0.066) |
| high | -0.244*** | -0.244*** | -0.244*** | -0.244*** | 0.595*** | 0.467*** | 0.420*** | 0.406*** |
| | (0.057) | (0.057) | (0.057) | (0.057) | (0.093) | (0.071) | (0.063) | (0.069) |
| Constant | 2.388*** | 1.043*** | -0.503*** | -1.880*** | 2.141*** | 1.013*** | -0.681*** | -1.728*** |
| | (0.154) | (0.123) | (0.104) | (0.085) | (0.116) | (0.104) | (0.093) | (0.090) |
| Observations | 6,274 | 6,274 | 6,274 | 6,274 | 17,258 | 17,258 | 17,258 | 17,258 |
| **Social activity** | | | | | | | | |
| Age range (ref. less than 24 years old) | | | | | | | | |
| 25-31 years old | -0.275*** | -0.275*** | -0.275*** | -0.275*** | 0.152** | 0.418*** | 0.457*** | 0.703*** |
| | (0.065) | (0.065) | (0.065) | (0.065) | (0.074) | (0.064) | (0.060) | (0.063) |
| 32-49 years old | -1.286*** | -1.184*** | -0.930*** | -0.829*** | -0.542*** | -0.290*** | -0.116** | 0.256*** |
| | (0.120) | (0.094) | (0.062) | (0.111) | (0.077) | (0.062) | (0.053) | (0.054) |
| more than 50 years old | -2.141*** | -1.877*** | -1.400*** | -1.045*** | -1.153*** | -0.774*** | -0.536*** | -0.130 |
| | (0.142) | (0.154) | (0.139) | (0.175) | (0.105) | (0.089) | (0.077) | (0.079) |
| Female (ref. male) | -0.053 | -0.053 | -0.053 | -0.053 | 0.316*** | 0.228*** | 0.098*** | 0.018 |
| | (0.044) | (0.044) | (0.044) | (0.044) | (0.043) | (0.047) | (0.036) | (0.037) |
| Urban (ref. rural-living) | 0.269 | 0.202** | 0.026 | 0.047 | 0.174*** | 0.174*** | 0.174*** | 0.174*** |
| | (0.169) | (0.083) | (0.065) | (0.081) | (0.038) | (0.038) | (0.038) | (0.038) |
| ISEI (ref. low) | | | | | | | | |
| lower middle | 0.147*** | 0.147*** | 0.147*** | 0.147*** | 0.489*** | 0.478*** | 0.342*** | 0.376*** |
| | (0.050) | (0.050) | (0.050) | (0.050) | (0.084) | (0.075) | (0.055) | (0.047) |
| middle | 0.245* | 0.080 | -0.014 | 0.018 | 0.842*** | 0.881*** | 0.684*** | 0.686*** |
| | (0.126) | (0.105) | (0.101) | (0.123) | (0.057) | (0.068) | (0.064) | (0.058) |

| | | | | | | | | |
|---|---|---|---|---|---|---|---|---|
| high | 0.255** | 0.084 | -0.017 | -0.197** | 1.198*** | 1.105*** | 0.924*** | 0.815*** |
| | (0.114) | (0.097) | (0.083) | (0.096) | (0.085) | (0.061) | (0.060) | (0.050) |
| Constant | 2.374*** | 1.456*** | 0.143*** | -1.450*** | 1.729*** | 1.028*** | -0.140** | -1.187*** |
| | (0.138) | (0.080) | (0.047) | (0.043) | (0.070) | (0.057) | (0.055) | (0.060) |
| Observations | 6,269 | 6,269 | 6,269 | 6,269 | 17,257 | 17,257 | 17,257 | 17,257 |

*Note* Robust standard errors in parentheses, *** p<0.01, ** p<0.05, * p<0.10. Four panels of internet activity evaluation included 1st panel (category 1 versus categories 2, 3, 4, and 5); 2nd panel (categories 1 and 2 versus categories 3, 4, and 5); 3rd panel (categories 1, 2, and 3 versus categories 4 and 5); and 4th panel (categories 1, 2, 3, and 4 versus category 5).

**Table S3.** Results of the generalised partial proportional odds model of cohorts predicting the importance of the online activities in the 2010 and 2018 panels.

| **Panel** | | **2010** | | | | **2018** | | |
|---|---|---|---|---|---|---|---|---|
| **Evaluations** | **1** | **2** | **3** | **4** | **1** | **2** | **3** | **4** |
| | | | | **Studying activity** | | | | |
| Age cohorts (ref. 1987-1994) | | | | | | | | |
| 1979-1986 | -0.010 | -0.234*** | -0.234** | 0.053 | 0.153* | 0.153** | 0.005 | 0.101 |
| | (0.195) | (0.085) | (0.098) | (0.150) | (0.089) | (0.074) | (0.066) | (0.076) |
| 1960-1978 | -0.482*** | -0.397*** | -0.142 | 0.237* | 0.099 | 0.099 | 0.099 | 0.099 |
| | (0.153) | (0.110) | (0.119) | (0.132) | (0.079) | (0.079) | (0.079) | (0.079) |
| Before 1960 | -1.155*** | -0.744*** | -0.223 | 0.127 | -0.165 | -0.165 | -0.165 | -0.165 |
| | (0.317) | (0.193) | (0.194) | (0.230) | (0.147) | (0.147) | (0.147) | (0.147) |
| | 0.097 | 0.097 | 0.097 | 0.097 | 0.065 | 0.065 | 0.065 | 0.065 |
| Female (ref. male) | (0.066) | (0.066) | (0.066) | (0.066) | (0.050) | (0.050) | (0.050) | (0.050) |
| Education (ref. less than middle school) | | | | | | | | |
| high school | 0.916*** | 0.582*** | 0.415*** | 0.125 | 0.636*** | 0.636*** | 0.636*** | 0.636*** |
| | (0.155) | (0.097) | (0.063) | (0.088) | (0.098) | (0.098) | (0.098) | (0.098) |
| > high school | 1.966*** | 1.231*** | 0.800*** | 0.381*** | 1.858*** | 1.815*** | 1.429*** | 1.141*** |
| | (0.268) | (0.147) | (0.090) | (0.133) | (0.132) | (0.120) | (0.108) | (0.090) |
| Urban (ref. rural- | 0.164** | 0.164** | 0.164** | 0.164** | -0.099 | -0.099 | -0.099 | -0.099 |
| living) | (0.081) | (0.081) | (0.081) | (0.081) | (0.115) | (0.115) | (0.115) | (0.115) |
| Income status (ref. low) | | | | | | | | |
| lower middle | -0.201 | -0.201 | -0.201 | -0.201 | -0.027 | -0.027 | -0.027 | -0.027 |
| | (0.123) | (0.123) | (0.123) | (0.123) | (0.182) | (0.182) | (0.182) | (0.182) |
| middle | -0.269*** | -0.269*** | -0.269*** | -0.269*** | 0.135 | 0.135 | 0.135 | 0.135 |
| | (0.082) | (0.082) | (0.082) | (0.082) | (0.126) | (0.126) | (0.126) | (0.126) |
| high | -0.359* | -0.359* | -0.359* | -0.359* | 0.221* | 0.221* | 0.221* | 0.221* |
| | (0.191) | (0.191) | (0.191) | (0.191) | (0.122) | (0.122) | (0.122) | (0.122) |
| Social status (ref. low) | | | | | | | | |
| lower middle | 0.466** | 0.126 | 0.041 | -0.355* | 0.406** | 0.289* | 0.234 | -0.002 |
| | (0.209) | (0.118) | (0.175) | (0.208) | (0.166) | (0.149) | (0.194) | (0.148) |
| middle | 0.714*** | 0.571*** | 0.303* | -0.111 | 0.298* | 0.275 | 0.213 | 0.035 |
| | (0.189) | (0.115) | (0.175) | (0.207) | (0.173) | (0.177) | (0.179) | (0.200) |
| high | 0.936*** | 0.920*** | 0.891*** | 0.298 | 0.401** | 0.401** | 0.401** | 0.401** |
| | (0.306) | (0.142) | (0.161) | (0.216) | (0.179) | (0.179) | (0.179) | (0.179) |
| Constant | 1.568*** | 0.764*** | -0.515*** | -1.601*** | -0.534*** | -0.642*** | -1.170*** | -1.680*** |
| | (0.209) | (0.139) | (0.164) | (0.181) | (0.191) | (0.187) | (0.172) | (0.203) |
| Observations | 3,040 | 3,040 | 3,040 | 3,040 | 3,003 | 3,003 | 3,003 | 3,003 |
| Pseudo R square | 0.0289 | 0.0289 | 0.0289 | 0.0289 | 0.0536 | 0.0536 | 0.0536 | 0.0536 |
| | | | | **Entertainment activity** | | | | |
| Age cohorts (ref. 1987-1994) | | | | | | | | |
| 1979-1986 | -0.223** | -0.223** | -0.223** | -0.223** | -0.263** | -0.263** | -0.263** | -0.263** |
| | (0.111) | (0.111) | (0.111) | (0.111) | (0.104) | (0.104) | (0.104) | (0.104) |

| | | | | | | | | |
|---|---|---|---|---|---|---|---|---|
| 1960-1978 | -0.983*** | -0.743*** | -0.545*** | -0.355** | -1.030*** | -0.933*** | -0.659*** | -0.538*** |
| | (0.151) | (0.111) | (0.131) | (0.142) | (0.162) | (0.135) | (0.091) | (0.101) |
| Before 1960 | -1.461*** | -1.075*** | -0.961*** | -0.166 | -1.482*** | -1.252*** | -0.707*** | -0.664** |
| | (0.187) | (0.154) | (0.137) | (0.156) | (0.217) | (0.188) | (0.138) | (0.260) |
| | -0.072 | 0.009 | -0.173*** | -0.177* | 0.345*** | 0.345*** | 0.345*** | 0.345*** |
| Female (ref. male) | (0.169) | (0.074) | (0.058) | (0.099) | (0.053) | (0.053) | (0.053) | (0.053) |
| Education (ref. less than middle school) | | | | | | | | |
| high school | 0.019 | 0.023 | 0.018 | -0.276*** | 0.052 | 0.052 | 0.052 | 0.052 |
| | (0.114) | (0.080) | (0.100) | (0.104) | (0.080) | (0.080) | (0.080) | (0.080) |
| > high school | -0.052 | -0.052 | -0.052 | -0.052 | 0.424*** | 0.070 | 0.180*** | 0.130 |
| | (0.100) | (0.100) | (0.100) | (0.100) | (0.137) | (0.093) | (0.063) | (0.092) |
| Urban (ref. rural- | 0.416** | 0.435*** | 0.468*** | 0.243* | 0.185 | 0.185 | 0.185 | 0.185 |
| living) | (0.183) | (0.114) | (0.082) | (0.128) | (0.126) | (0.126) | (0.126) | (0.126) |
| Income status (ref. low) | | | | | | | | |
| lower middle | 0.211** | 0.211** | 0.211** | 0.211** | 0.111 | 0.111 | 0.111 | 0.111 |
| | (0.097) | (0.097) | (0.097) | (0.097) | (0.137) | (0.137) | (0.137) | (0.137) |
| middle | 0.273*** | 0.273*** | 0.273*** | 0.273*** | 0.198 | 0.344*** | 0.050 | -0.006 |
| | (0.081) | (0.081) | (0.081) | (0.081) | (0.125) | (0.123) | (0.136) | (0.122) |
| high | 0.667*** | 0.667*** | 0.667*** | 0.667*** | 0.150 | 0.150 | 0.150 | 0.150 |
| | (0.141) | (0.141) | (0.141) | (0.141) | (0.151) | (0.151) | (0.151) | (0.151) |
| Social status (ref. low) | | | | | | | | |
| lower middle | 0.191 | 0.054 | -0.026 | -0.376*** | 0.171 | -0.218* | -0.402*** | -0.210 |
| | (0.193) | (0.162) | (0.116) | (0.131) | (0.185) | (0.112) | (0.146) | (0.164) |
| middle | 0.547*** | 0.213 | 0.067 | -0.289* | -0.042 | -0.042 | -0.042 | -0.042 |
| | (0.203) | (0.145) | (0.146) | (0.149) | (0.119) | (0.119) | (0.119) | (0.119) |
| high | 0.054 | -0.036 | 0.239 | -0.325 | -0.004 | -0.004 | -0.004 | -0.004 |
| | (0.184) | (0.181) | (0.167) | (0.214) | (0.152) | (0.152) | (0.152) | (0.152) |
| Constant | 2.019*** | 0.826*** | -0.656*** | -1.410*** | 2.076*** | 1.311*** | -0.150 | -1.023*** |
| | (0.268) | (0.173) | (0.156) | (0.140) | (0.184) | (0.164) | (0.180) | (0.174) |
| Observations | 3,041 | 3,041 | 3,041 | 3,041 | 3,003 | 3,003 | 3,003 | 3,003 |
| Pseudo R square | 0.0188 | 0.0188 | 0.0188 | 0.0188 | 0.0247 | 0.0247 | 0.0247 | 0.0247 |
| **Social activity** | | | | | | | | |
| Age cohorts (ref. 1987-1994) | | | | | | | | |
| 1979-1986 | -0.401*** | -0.401*** | -0.401*** | -0.401*** | -0.096 | -0.096 | -0.096 | -0.096 |
| | (0.097) | (0.097) | (0.097) | (0.097) | (0.090) | (0.090) | (0.090) | (0.090) |
| 1960-1978 | -1.082*** | -1.082*** | -1.082*** | -1.082*** | -0.545*** | -0.545*** | -0.545*** | -0.545*** |
| | (0.113) | (0.113) | (0.113) | (0.113) | (0.078) | (0.078) | (0.078) | (0.078) |
| Before 1960 | -1.967*** | -1.781*** | -1.546*** | -1.165*** | -1.709*** | -1.437*** | -1.230*** | -1.210*** |
| | (0.206) | (0.193) | (0.192) | (0.258) | (0.172) | (0.197) | (0.133) | (0.157) |
| | -0.110* | -0.110* | -0.110* | -0.110* | 0.856*** | 0.696*** | 0.466*** | 0.346*** |
| Female (ref. male) | (0.063) | (0.063) | (0.063) | (0.063) | (0.223) | (0.154) | (0.086) | (0.066) |
| Education (ref. less than middle school) | | | | | | | | |
| high school | 0.556*** | 0.256*** | 0.136* | 0.106 | 0.239*** | 0.239*** | 0.239*** | 0.239*** |
| | (0.106) | (0.082) | (0.078) | (0.143) | (0.081) | (0.081) | (0.081) | (0.081) |

|  |  |  |  |  |  |  |  |  |
|---|---|---|---|---|---|---|---|---|
| > high school | 0.272** | 0.041 | -0.067 | -0.086 | 1.106*** | 0.838*** | 0.618*** | 0.511*** |
|  | (0.115) | (0.105) | (0.085) | (0.142) | (0.188) | (0.137) | (0.113) | (0.104) |
| Urban (ref. rural- | 0.088 | 0.088 | 0.088 | 0.088 | -0.105 | -0.105 | -0.105 | -0.105 |
| living) | (0.112) | (0.112) | (0.112) | (0.112) | (0.098) | (0.098) | (0.098) | (0.098) |
| Income status (ref. low) |  |  |  |  |  |  |  |  |
| lower middle | -0.020 | -0.020 | -0.020 | -0.020 | 0.272* | 0.272* | 0.272* | 0.272* |
|  | (0.109) | (0.109) | (0.109) | (0.109) | (0.160) | (0.160) | (0.160) | (0.160) |
| middle | 0.342*** | 0.109 | -0.056 | 0.001 | 0.464*** | 0.464*** | 0.464*** | 0.464*** |
|  | (0.122) | (0.089) | (0.070) | (0.106) | (0.148) | (0.148) | (0.148) | (0.148) |
| high | 1.072*** | 0.441** | 0.201 | -0.036 | 0.595*** | 0.595*** | 0.595*** | 0.595*** |
|  | (0.284) | (0.196) | (0.135) | (0.256) | (0.162) | (0.162) | (0.162) | (0.162) |
| Social status (ref. low) |  |  |  |  |  |  |  |  |
| lower middle | 0.143 | -0.166 | 0.031 | 0.021 | 0.624** | -0.034 | -0.036 | -0.302* |
|  | (0.175) | (0.215) | (0.178) | (0.210) | (0.274) | (0.268) | (0.163) | (0.164) |
| middle | 0.244 | 0.244 | 0.244 | 0.244 | 0.224 | -0.055 | -0.118 | -0.413** |
|  | (0.149) | (0.149) | (0.149) | (0.149) | (0.245) | (0.243) | (0.175) | (0.189) |
| high | 0.078 | 0.260 | 0.628*** | 0.526*** | -0.066 | -0.350 | -0.032 | -0.280* |
|  | (0.287) | (0.224) | (0.165) | (0.192) | (0.301) | (0.313) | (0.199) | (0.165) |
| Constant | 2.030*** | 1.342*** | 0.023 | -1.529*** | 1.719*** | 1.571*** | 0.353 | -0.201 |
|  | (0.255) | (0.216) | (0.159) | (0.130) | (0.367) | (0.342) | (0.222) | (0.169) |
| Observations | 3,040 | 3,040 | 3,040 | 3,040 | 3,003 | 3,003 | 3,003 | 3,003 |
| Pseudo R square | 0.0343 | 0.0343 | 0.0343 | 0.0343 | 0.0381 | 0.0381 | 0.0381 | 0.0381 |
| **Evaluations** | **1** | **2** | **3** |  | **1** | **2** | **3** |  |
|  |  |  |  | **Working activity** |  |  |  |  |
| Age cohorts (ref. 1987-1994) |  |  |  |  |  |  |  |  |
| 1979-1986 | -0.061 | -0.061 | -0.061 |  | 0.173* | 0.173* | 0.173* |  |
|  | (0.077) | (0.077) | (0.077) |  | (0.098) | (0.098) | (0.098) |  |
| 1960-1978 | -0.167* | -0.273*** | -0.052 |  | -0.086 | -0.086 | -0.086 |  |
|  | (0.093) | (0.076) | (0.111) |  | (0.114) | (0.114) | (0.114) |  |
| Before 1960 | -0.764*** | -0.764*** | -0.764*** |  | -2.295*** | -2.295*** | -2.295*** |  |
|  | (0.170) | (0.170) | (0.170) |  | (0.322) | (0.322) | (0.322) |  |
|  | 0.073 | 0.073 | 0.073 |  | 0.026 | 0.026 | 0.026 |  |
| Female (ref. male) | (0.059) | (0.059) | (0.059) |  | (0.077) | (0.077) | (0.077) |  |
| Education (ref. less than middle school) |  |  |  |  |  |  |  |  |
| high school | 0.602*** | 0.494*** | 0.326*** |  | 0.667*** | 0.667*** | 0.667*** |  |
|  | (0.089) | (0.062) | (0.097) |  | (0.095) | (0.095) | (0.095) |  |
| > high school | 1.118*** | 1.039*** | 0.755*** |  | 2.010*** | 1.857*** | 1.576*** |  |
|  | (0.121) | (0.072) | (0.097) |  | (0.092) | (0.094) | (0.102) |  |
| Urban (ref. rural- | 0.098 | 0.098 | 0.098 |  | 0.248*** | 0.248*** | 0.248*** |  |
| living) | (0.076) | (0.076) | (0.076) |  | (0.093) | (0.093) | (0.093) |  |
| Income status (ref. low) |  |  |  |  |  |  |  |  |
| lower middle | 0.288** | 0.288** | 0.288** |  | 0.818*** | 0.672*** | 0.479*** |  |
|  | (0.122) | (0.122) | (0.122) |  | (0.150) | (0.180) | (0.186) |  |
| middle | 0.485*** | 0.485*** | 0.485*** |  | 1.098*** | 1.012*** | 0.717*** |  |

| | | | | | | |
|---|---|---|---|---|---|---|
| | (0.098) | (0.098) | (0.098) | (0.169) | (0.173) | (0.172) |
| high | 0.526** | 0.526** | 0.526** | 0.910*** | 0.872*** | 0.657*** |
| | (0.240) | (0.240) | (0.240) | (0.166) | (0.149) | (0.185) |
| Social status (ref. low) | | | | | | |
| lower middle | 0.001 | -0.156 | -0.553*** | -0.216 | -0.216 | -0.216 |
| | (0.118) | (0.160) | (0.202) | (0.178) | (0.178) | (0.178) |
| middle | 0.161 | 0.039 | -0.381* | -0.193 | -0.193 | -0.193 |
| | (0.109) | (0.149) | (0.198) | (0.160) | (0.160) | (0.160) |
| high | 0.630*** | 0.552** | -0.097 | -0.077 | -0.077 | -0.077 |
| | (0.148) | (0.218) | (0.250) | (0.162) | (0.162) | (0.162) |
| Constant | -0.079 | -0.862*** | -1.573*** | -1.464*** | -1.770*** | -1.919*** |
| | (0.100) | (0.129) | (0.181) | (0.155) | (0.166) | (0.195) |
| Observations | 3,036 | 3,036 | 3,036 | 3,003 | 3,003 | 3,003 |
| Pseudo R square | 0.0349 | 0.0349 | 0.0349 | 0.111 | 0.111 | 0.111 |

*Note* Robust standard errors in parentheses, *** p<0.01, ** p<0.05, * p<0.10. Four panels of studying, entertainment and social activity evaluation included 1st panel (category 1 versus categories 2, 3, 4, and 5); 2nd panel (categories 1 and 2 versus categories 3, 4, and 5); 3rd panel (categories 1, 2, and 3 versus categories 4 and 5); and 4th panel (categories 1, 2, 3, and 4 versus category 5). Three panels of working activity evaluation included 1st panel (category 1 versus categories 2, 3, and 4); 2nd panel (categories 1 and 2 versus categories 3 and 4); 3rd panel (categories 1, 2, and 3 versus category 4).
